# Sensing of Volatile Organic Compounds by Haller’s Structure in Ixodidae Tick: Electroscutumography and Olfactometric Bioassay

**DOI:** 10.3390/bios15060358

**Published:** 2025-06-04

**Authors:** Alivia Mandal, Bishwajeet Paul, Biswanath Bhowmik, Raja Reddy Gundreddy, Adolat U. Mirzaieva, Kakali Bhadra

**Affiliations:** 1 Department of Zoology, University of Kalyani, Nadia 741235, India; aliviamandal99@gmail.com (A.M.); bbklec@gmail.com (B.B.); 2 Division of Entomology, ICAR-Indian Agricultural Research Institute, Pusa Campus, New Delhi 110012, India; bishwajeet_paul2011@yahoo.com (B.P.); rajareddygundreddy422@gmail.com (R.R.G.); 3 Institute of Zoology of the Republic of Uzbekistan, Tashkent 100053, Uzbekistan; mirzaieva_a.u@mail.ru

**Keywords:** volatile organic compounds, Haller’s organ, *Haemaphysalis darjeeling*, microanatomy, olfactometer, tick electrophysiology

## Abstract

**Background:** Chemosensation in ticks opens a novel and unique field for scientific research. This study highlights ticks’ chemosensory system to comprehend its host-searching behavior and other integrated chemistry and biology involving Haller’s structure. **Methodology:** This study combines microanatomical, electrophysiological, and behavioral experiments to investigate the role of Haller’s organ in adult ticks in response to different classes of organic compounds. **Results:** We showed the microscopic anatomy of Haller’s organ in *Haemaphysalis darjeeling*, present at the terminal segment of the first pair of appendages. Haller’s structure serves a vital function in perceiving odor. The electrophysiological activity of adult ticks to different classes of organic compounds via electroscutumography was explored at five different concentrations: *w*/*v* 0.001, 0.01, 0.1, 1.0, and 2.0%. Among 55 organic compounds, moderate to high stimulation was recorded with pyruvate (13.28 mv at 2%), ammonia (12.26 mv at 2%), benzoic acid (1.99 mv at 0.001%), isobutyric acid (1.39 mv at 0.001%), 2,6-dichlorophenol (1.34 mv at 0.001%), p-Tolualdehyde (1.26 mv at 2%), tetradecane (1.23 mv at 2%), docosane (1.17 mv at 2%), citronellal (1.13 mv at 0.1%), isopropyl acetate (1.05 mv at 0.01%), cyclohexanol (1.03 mv at 2%), 1-octane-3-ol (1.02 mv at 2%), and 1-octanol (1.01 mv at 0.001%). Olfactometric bioassays at *w*/*v* 2.0% concentration further confirmed that ammonia, pyruvate, 1-octane-3-ol, hematin porcine, p-Tolualdehyde, methyl salicylate, uric acid, tetradecane, carbon dioxide, propanoic acid, 3-hexanol, hexanoic acid, adenine, 2,6-dichlorophenol, hexadecane, heptanoic acid, pentanoic acid, octadecane, guanine, and nonanoic acid acted as strong attractants, while citronellal, eugenol, butyric acid, geraniol, benzaldehyde, and tiglic aldehyde showed an active repellent effect against the tick species. **Conclusions:** This investigation provides knowledge of the olfactory sensilla of Haller’s structure as biosensors behind tick olfaction and the possibility for chemical detection of diverse attractants and repellents for future development of anti-tick compounds.

## 1. Introduction

Synthetic acaricides like organophosphates, synthetic pyrethroids, macrocyclic lactones, chlorinated hydrocarbons, formamidines, carbamates, etc. are often applied in controlling tick populations [1]; however, intensive use of these available chemical ectoparasiticides has contaminated the environment and created populations of ticks that are resistant to several acaricides, which is extremely concerning [2,3]. Ticks can develop resistance against acaricides in various ways, including alterations in amino acid sequence that result in structural changes in the acaricide target molecules, detoxification of metabolic pathways, and decreased acaricide penetration through the tick body outer layer [2,3]. To mitigate these effects, great emphasis has been placed on the development of alternative, ecologically friendly parasite control techniques that have a lower probability of developing resistance [4,5,6,7,8]. The current research intends to screen out eco-friendly and target-specific anti-tick compounds based on tick electrophysiological and olfactometric bioassays to give an effective tool for managing these resistant ticks.

An important feature, olfactory chemosensation, plays a significant role in controlling essential behaviors of living organisms. When compared to insect chemosensory systems, tick chemosensation creates a new and distinct area for scientific study [9,10,11,12]. Not much information is available about this species’ chemosensory system. Ticks, as arthropod vectors, are hematophagous ectoparasites that spread most diseases they carry, and the incidence of tick-borne diseases is rising because of population growth, globalization, and climate change [9,13,14,15]. The development of ticks requires blood from feeding on a host for molting, metamorphosis, sexual maturity, and reproduction. Blood feeding offers a chance for disease transmission between the host and the tick [16,17]. Ticks use semiochemical signals that serve as attractants/deterrents/repellents to locate their hosts [9]. *Haemaphysalis darjeeling*, which is reported to be one of the most prevalent species in the present locality from the host species *Bos taurus taurus* (Lin) (Family: Bovidae), domesticated cattle, has seasonal fluctuations in their prevalence, with peak periods throughout the rainy season from June to August [18].

Electrophysiology offers a very effective and efficient way to evaluate chemical signaling because the nervous system predominantly employs electrical potentials to transfer information. Ticks are a special and intriguing species for studying the detection of chemical cues from the host with the help of electrophysiological techniques because of the absence of antennae, the presence of few olfactory sensilla, the development of Haller’s organ, and the presence of multimodal sensilla on mouthparts [11,19,20,21]. Earlier, few studies have examined how Ixodidae ticks like *Amblyomma americanum* (Linn), *A. sculptum*, *A. maculatum* (Koch), *Dermacentor variabilis* (Say), *Ixodes ricinus* (Linn), and *I. scapularis* react to various attractant cues of appropriate hosts [22,23,24,25,26,27,28]. Researchers applied a conventional electrophysiological technique to investigate the role of neurons sensitive to 2,6-dichlorophenol on *A. americanum* (Linn) [29]. Further investigation showed an innovative technique by inserting a glass capillary electrode in the synganglion to correlate the behavior of ticks in relation to odors [30]. Several behavioral bioassays were used to confirm the attractant and repellent properties of both synthetic and natural compounds, enabling the possible bio-active components for powerful tick control [6,7,8,11,31,32,33,34,35,36,37,38,39]. Several non-volatile topical applications of crude, aqueous, and alcoholic plant extracts have been reported to show acaricidal activity [11,31,32,33,34,35,36,37,38,39].

Haller’s structure, which is a unique organ in ticks, includes a set of different types of chemoreceptive sensilla analogous to insect antennae [11,40]. Tick olfaction involves several chemosensory-related compounds that have been identified by Haller’s structure-targeted transcriptome and proteome analyses [41]. Several findings have established the role of olfaction in ticks’ Haller’s organ, which directs them in locating their hosts [11,40,41]. Apart from its olfactory detection, Haller’s organ can also sense infrared light and radiant heat, as reported in different species of ticks [42,43]. Scanning electron microscopy further showed significant morphological variations among Haller’s organs of different genera, and sexual dimorphism was observed in *Dermacentor variabilis* ticks [44,45]. However, this appears to be in opposition to the fact that species do not differ from one another in terms of Haller’s organ structure [46]. It was even hypothesized that changes in Haller’s organ structure and variations in its life history patterns are related, but the association cannot be verified based on current data [47]. Haller’s structure has been examined in detail in many groups of tick species [47,48,49]. Different types of receptors found in the Haller’s capsule respond differently to different classes of compounds [12,50]. One of the few sensilla, like the wall-pore olfactory sensillum and sensillum DI.1, present in the capsule of the Haller’s structure, has been the focus of single sensillum recording (SSR), which has been carried out in *A. variegatum*, *Ixodes ricinus*, and *A. cajennense* (Fab) [49,50,51]. However, the precise structure and function of Haller’s organ in *H. darjeeling* are still largely unknown.

In this investigation, the electrophysiological response of the chemosensory system to different categories of volatile organic compounds (VOCs) using an innovative electroscutumography (ESG) technique was examined in adult female *H. darjeeling* ticks. Different organic compounds of varied moieties were sorted out for the electrophysiological analysis. Randomly selected compounds from plant-based volatile organic compounds were used. Moreover, the behavioral study of this tick species was further highlighted using olfactometric bioassays with a multichannel [52,53] and Y-tube olfactometer [54] for the ultimate selection and screening of active repellants and attractants in *H. darjeeling* for future formulations as eco-friendly compounds. This work enhances the knowledge of chemoreception in the tick species, possible interactions with VOCs influencing tick host-finding behavior, and the repellent nature of the target-specific compounds.

## 2. Materials and Methods

### 2.1. Collection and Culture of Ticks

Fully engorged adult *Haemaphysalis darjeeling* ticks were collected under clinical supervision by a veterinarian from the naturally infested hosts of the species *Bos taurus taurus* (Holstein Frisian crossbreed), aged 2–3 years, the predominant cow population in Nadia district, West Bengal (Lat: 22.9605° N; Long: 88.5674° E). The collected specimens were incubated in constant darkness (T = 27 ± 2 °C, RH > 60 ± 5%) under laboratory conditions to develop a colony. Immature stages were fed on goat blood through a membrane feeding system. Ticks that had been fed and had just molted were kept in the same environment. Unfed, naïve, host-seeking *H. darjeeling* adult ticks (males and females) were used in the electrophysiological recordings and olfactometric assay. Each tick was used only once in a single experiment and was later discarded by wrapping it in a PP plastic bag, which was handed over to Biomedical Waste Management PVT. Ltd., New Delhi, India.

### 2.2. Stimulants

Table 1 provides the list of the various organic compounds used in the electroscutumography (ESG) and olfactometric bioassays, their purity, and commercial vendors. The compounds were selected based on prior research that reported their behavioral influence on different tick species [23,27,28,49,54]. Additionally, some common VOCs obtained from plants were also included in the electrophysiological study. To dissolve the organic compounds, paraffin liquid light, procured from Hi-Media, Maharashtra, India, was employed.

### 2.3. Electrophysiological Assay by ESG Recording

Unfed adult ticks were used in the experiments at 2–3 months after molting. Before starting the ESG, unfed adult ticks were placed outside the incubator for 15 min to allow them to adapt to ambient temperature. A single tick was placed head-first on a glass Petri plate containing a 5 mm layer of wax, facing toward the stimulus airflow. An entomological pin measuring 0.30 mm was used to attach the tick body’s abdomen to the wax layer of the Petri dish, rendering the specimen immobile. An entomological pin was used to make two tiny piercings, one at the scutum in the synganglion [20,28], and another at the alloscutum position (Figure S1), which were viewed using a digital zoom microscope (Euromex Nexius, Holland). Recording and ground electrodes made of silver wire within glass tubes were electrolytically sharpened to 1 µm and saturated in 0.1 N NaOH solution to maintain the continuous conductivity of the silver wire. The ground electrode was placed inside the alloscutum, and the recording electrode was similarly inserted into the scutum using the incision that was made earlier. The tick preparation received a continuous humidified airflow (0.5 L/min) through a 30 cm long glass air delivery tube with a single 2 mm diameter hole situated 10 cm from the output. IDAC-2 (Syntech, Hilversum, the Netherlands) technology, was used to gather and enhance electrical potential changes. The overall amplification was performed ten times. The ESG amplitude was estimated using customized ESG software (version 2.6c, 1998, Syntech, Hilversum, The Netherlands). A 20 μL individual sample, dissolved in paraffin liquid light, was applied to a 2 × 5 cm piece of filter paper (Whatman^®^, quantitative filter paper, Merck) and dried in a fume hood for 10 min. Both forms of stimuli (attractant and repellent) were loaded separately on the same cartridge. Cartridges were loaded individually into 3 mL glass Pasteur pipettes with 1000 μL pipette tips. Stimulus was provided as an air puff for 0.5 s while the tip of the Pasteur pipette was put through the opening in the air delivery tube. Stimuli were delivered at an interval of 1 min to allow the olfactory system to recover to baseline rates between puffs. The ESG peak was interpreted in millivolts (mv). These values aided in determining the relative mean amplitudes in percentages, mean values, standard error, and standard deviation.

### 2.4. Statistical Analysis

Social Science Statistics software (2022) was used for statistical analysis. For dosage–response investigations, the trials were carried out in a completely randomized design with five treatments (*w*/*v* 0.001, 0.01, 0.1, 1, and 2% concentrations) and ten replications each. ESG values were collected and plotted at five different concentrations against 55 chemicals. The data were then subjected to square root transformation for normalization to stabilize the variance and bring the mean to around 1, reducing noise in the dataset and improving the fit to a normal distribution so that the statistical analysis could be performed with ease. One-way ANOVA was performed to compare the mean differences (A) among the five concentrations and (B) between two different conditions, i.e., ticks with intact legs and those after surgical removal of Haller’s organ, across all five concentrations.

### 2.5. Microscopic Analysis

#### 2.5.1. SEM

SEM investigations were conducted on the adult phases of 12 (3 male + 9 female) specimens. Ticks were euthanized by freezing at −80 °C for at least 2 h, then removed from the freezer, cleaned thoroughly with 70% ethanol, and kept in fresh 70% ethanol in a 1.5 μL microcentrifuge tube until imaging was conducted. Extracellular debris was removed carefully. The specimens were fixed with 2% glutaraldehyde solution from Sigma Aldrich, India, for 12 h at 4 °C. After overnight fixation, the specimens were rinsed twice with phosphate-buffered saline and double-distilled water for 15 min intervals. Specimens were rinsed repeatedly in the buffer, dehydrated in ascending grades of ethanol (30–100%) for 15 min each, air-dried, placed in a vacuum desiccator for 48 h, and then kept on aluminum stubs using double-sided sticky carbon tape. Specimens were coated with gold–palladium sputter (10–15 nm thickness, Polaron-SC7620). Finally, the surface topography of the specimen was examined under SEM (ZEISS EVO 18, operated at 20 kV) at the University of Kalyani, S.N.B.I Centre, Kalyani, India.

#### 2.5.2. Light Microscope

For the preparation of a light microscopy sample, Haller’s organ, located at the first tarsal segment of the forelegs, was surgically removed from a freshly collected tick specimen. After dissection, fixation of the structure was carried out with 2% paraformaldehyde solution (Sigma Aldrich), and the sample was dehydrated with gradually increasing concentrations of alcohol. The next step was clearing, wherein paraffin wax was allowed to infiltrate by eliminating the alcohol using xylene. Melted paraffin wax formed a large block around the tissue. After the solidification of the block, very thin tissue sections of 2 µm, in the form of ribbons, were made using a SACM 1090A Microtome from Western Electric & Scientific Works, India. Staining of the tissue sections was performed using hematoxylin and eosin stain from Sigma-Aldrich, India. Finally, the mounted slides were observed under a Zeiss Primo Star microscope (ZEISS EVO 18, Maharashtra, India, 2018) at 20× 40×, and 60× magnifications.

#### 2.5.3. TEM

The first tarsal segment of the forelegs of 20 (6 male and 14 female) *H. darjeeling* tick specimens (adult stage) was dissected and fixed in 2% glutaraldehyde solution for 6 h at 4 °C. After fixation, the specimens were rinsed with PBS to remove extracellular debris. After dehydration, the tissues were embedded with gradually increasing concentrations of liquid resin and cured into a hard block using heat. Polymerization of the resin blocks was performed in an oven at 50 °C for 24 h and then at 60 °C for 24 h. The hardened resin samples were cut for electron microscopy. Thin sections (400–700 Å) were prepared using a microtome (Technai Ultracut) and placed on 100-mesh naked honeycomb copper grids. Biological specimens are naturally not very electron-opaque, as they are composed of atoms with low atomic numbers, and the beam passes through them easily. To increase sample contrast, the sections were placed on numbered grids, stained with 0.5% uranyl acetate and 0.5% lead citrate (Sigma, Chennai, India), and examined under a high-voltage (120 kV) TEM (transmission electron microscope, Technai electron microscopes) from the All India Institute of Medical Sciences (AIIMS), New Delhi.

### 2.6. Olfactometry

Chemicals obtained from commercial vendors (Table S1) were employed in the olfactory bioassay to determine the attractant/repellent properties of the compounds in adult *H. darjeeling*. To ensure a better response, the test specimens were starved for four to five hours prior to the experiment.

#### 2.6.1. Multichannel Olfactometer

Figure S2 shows a schematic illustration of the detailed structure of the eight-arm olfactometer. A vacuum pump (DIAPHRAGM PUMP, BioSbe, India) with an air inlet tube generates a pressure of 10 psi. (A) shows the bottom shelf with a round-bottom flask (250 mL) containing water to generate humid airflow. (B) shows the middle shelf containing a conical flask (150 mL) with eight small channels. The conical flask is connected to the round bottom flask with a glass adaptor 6 cm in length, connected with a silicon tube (1 cm diameter). (C) shows the top shelf, which contains the olfactometry vessel with a 20 cm diameter with eight arms, each 3 cm in diameter and 15 cm in length. A tick was released in the center of the olfactometry chamber, where it could choose between one of eight odors placed in the eight different arms. The olfactometry vessel was connected to a second vacuum pump with an air outlet tube. The function of the air outlet pump was to ease the pressure generated by the humid air flow and maintain a unidirectional flow of air. The numbers (1, 2, 3, 4, 5, and 6) denoted in the schematic figure represent the sequence of events in the entire process. The whole apparatus was assembled on a 1 m × 1 m × 1.5 m high, three-tiered wooden cabinet. Openings on the top two shelves allowed connectivity between the different levels via silicon tubing. The lowest shelf (10 cm from the floor) held the humid air sources. A middle shelf (85 cm from the floor) helped in the release of the humid air through eight small arms connected by the silicon tube, whereas the top shelf (125 cm from the floor) carried the olfactometer vessel itself. All glass parts were custom-made by S.N. Enterprises (Delhi, India).

During the bioassay in the eight-arm olfactometer, different chemical compounds (*w*/*v* 2% each) were exposed in four arms of the olfactometry tube, and the other four tubes were left empty. A quantity of 20 µL of each compound was poured onto a piece of 1 cm × 1 cm filter paper, against which the specimen showed responses or stimulation using the ESG method. Filter paper, in olfactometry tubes, was placed in alternate arms of the olfactometer. This allowed the tick to flee to the empty tube if it found the chemical repulsive. For each experiment, we took four different chemicals, and, in each instance, we exposed only one specimen to the chemicals to avoid intraspecific semiochemical interference. Since, unlike insects, ticks are slow-moving, they were given 6 min after being placed in the vessel to adjust to the environment and make a decision as to which tube to enter. In order to obtain a positive response, the specimen needed to enter the chemical-laced tube and spend at least 2 min in the tube; conversely, for the response to be negative, the specimen needed to move into a chemical-laced tube and turn back immediately within a few seconds to enter the empty tube next to it. Specimens that did not seek either tube were considered non-responsive. For each set of four chemical compounds, 30 specimens were tested using the abovementioned experimental protocol, and the whole test was repeated 3 times. Thus, the mean value of responses to each compound was based on 90 ticks. The response was presented as the number of ticks that moved to the arm of the olfactometer.

Based on data from the multichannel olfactometric bioassay, binomial test analysis between positive and negative responses was performed, and a right-tailed distribution at a significance level of 0.05 was taken to determine whether the results were significant or just random occurrences.

#### 2.6.2. Y-Tube Olfactometer

The behaviors of adult *H. darjeeling* specimens toward different volatiles were further analyzed using a Y- or forked-shaped glass olfactometer (2.0 cm radius, 13 cm arms, 45° Y angle) [55]. Multichannel olfactometry was used to determine whether the specific chemical was an attractant or a repellent, and later, the Y-tube bioassay was used to determine how strong the attraction or repulsion was. A total of three replications (ten ticks/replication) were conducted for each compound. Thus, each treatment involved stimulation tests with 30 specimens to minimize statistical error. The average value of responses for each chemical (*w*/*v* 2% concentration) was calculated using a total of 90 ticks.

In the two-armed Y-tube assay, one arm was exposed to a 1 cm × 1 cm piece of chemically treated filter paper, and the other arm was left vacant/free. The specimen was placed in the free space of the Y tube. When the humid air passed (with a flow speed of 6 LPM) through the vacant and chemically treated tubes toward the specimen, it allowed the tick to select between the two arms, and the time taken by the specimen to move toward the chemically treated arm or the arm with the control solvent was measured. In order to obtain a positive or negative response, the specimen needed to spend at least 2 min in the respective tube. For each chemical, we exposed a single specimen at a time and tested a total of 10 samples for each test. We repeated the experiment 3 times, with a total of 30 specimens, to minimize statistical error.

The bioassays using the eight-arm and Y-tube olfactometers were carried out in a controlled laboratory environment (27 ± 2 °C, 60 ± 5% RH, and 70 lux). Prior to the final analysis, all data were converted using square root transformation. Both bioassays employed volatile chemicals obtained from commercial vendors to evaluate the attractive and repellent properties of the chemicals in adult ticks.

## 3. Results

### 3.1. Structural Anatomy of Haller’s Organ: SEM, Light Microscopy, and TEM Observations

**Figure 1:**
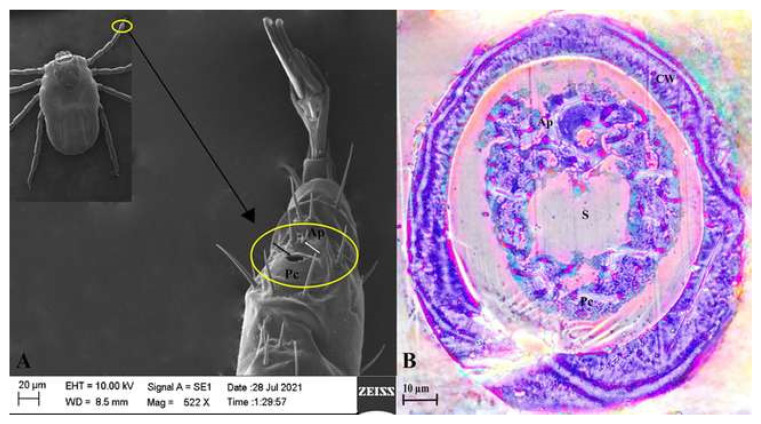
(**A**,**B**) Scanning electron micrograph and light microscopy images of Haller’s organ in *Haemaphysalis darjeeling* (CW: cuticular wall; Ap: anterior pit; Pc: proximal capsule; S: slit).

Haller’s organ of *Haemaphysalis darjeeling* was structurally characterized by scanning electron microscopy images [42,44,45,46,47,48]. SEM revealed the oval-shaped Haller’s structure of *H. darjeeling,* which is located on the tarsal segment of the foreleg. The entire Haller’s organ is 80 μm in length and 50 μm wide. Two distinct regions were observed: the anterior pit (Ap), with six sensilla, and the proximal capsule (Pc), with a single centrally located thick slit/opening (Figure 1A). The margin of the slit is irregular. The slit helps with communication with the outside. The six sensilla are close together and occupy an area of approximately 130 μ^2^. The slit is 12 μm long and 18 μm wide. Three of the six sensilla are medium in size, two are shorter than the other two, and one is somewhat longer. In comparison to the other five sensilla, the longer sensillum is thicker. One shorter sensilla has a thickness greater than the other. Therefore, the sensilla present in the anterior pit can be divided into three distinct categories, i.e., 1:3:2 (long: moderate: short), based on their length. Furthermore, the entire Haller’s organ is surrounded by several somewhat long tarsal spines that probably help in mechanoreception.

Under a light microscope, Haller’s structure further showed a thick outer cuticular surrounding (Figure 1B). The cuticular wall had a small number of pores on it. Beneath the cuticular wall, there was a relatively slender continuous muscle layer. The structure showed two distinct parts: the proximal capsule and the anterior pit. The section further showed a broad, centrally located, distinct slit or opening. Sensillum bases were visible in the anterior pit region, with two circular pores. Each sensillum base is surrounded by a separate layer of cuticle.

**Figure 2:**
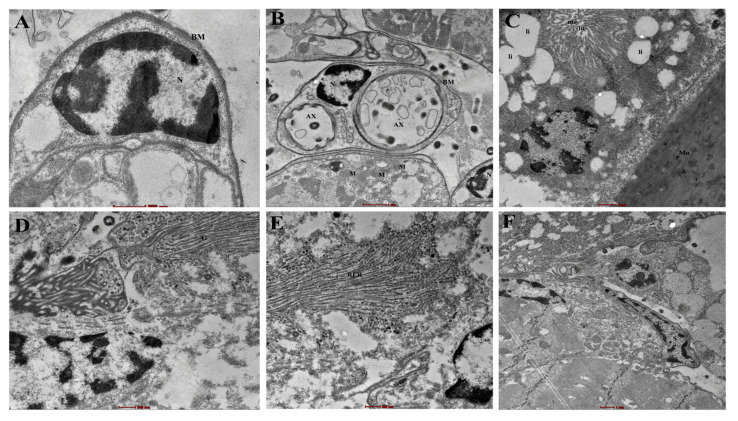
Ultrastructural details of Haller’s organ of *Haemaphysalis darjeeling*. Cellular structures showing (**A**) nucleus (N) situated at the base, (**B**) mitochondria (M), thin basement membrane (BM) of the cell, section of neurosecretory-type axon (AX) ×15,000, (**C**) microvilli (mv) with centrally placed lumen (lu), large lipid droplets (li), smooth muscle (Mu), (**D**) Golgi complex (Gol), (**E**) rough endoplasmic reticulum (RER), (**F**) longitudinal section of sensillum.

The structure also showed some fine-detail features, as observed under a transmission electron microscope (Figure 2A–F). Such structures include the sensillum base (S), cellular organelles, and details of the smooth muscle (Mu). Multicellular glands were visible underlying the proximal capsule of Haller’s organ. At a higher resolution, each gland cell was characterized by typical cell organelles, and inclusions like a large nucleus (N) situated at the base, mitochondria (M), microvilli (mv) with a centrally placed lumen (lu), large lipid droplets (li) in the cytoplasm, rough endoplasmic reticulum (RER), and Golgi-complex (Gol) were observed. The exterior of the gland is covered by a thin basement membrane (BM), which may also include a neurosecretory-type axon (AX).

### 3.2. ESG Dose–Response Studies in Haemaphysalis darjeeling

Unfed adult *H. darjeeling* ticks (N = 50 for each odorant) responded to 55 VOCs (volatile organic compounds) at five different concentrations (Section 2.4), evaluated using ESG spectra, with variations in response. Chemicals were randomly sorted out based on preliminary information, volatiles associated with vertebrates [23,27,28,51,55,56], compounds identified from the GC–MS profile from body swabs of cattle, and other common plant VOCs. Every test stimulus elicited ESG responses with a recognizable shape and a fairly consistent and repeatable time course. The raw data of a few stimulated odorants’ representative ESG spectra, recorded in millivolts, is shown in Figure S3. The mean ESG response of *H. darjeeling* at 0.001, 0.01, 0.1, 1, and 2% concentrations by paraffin liquid (standard) were 0.016 ± 0.003, 0.034 ± 0.004, 0.027 ± 0.003, 0.039 ± 0.007, and 0.045 ± 0.006 mv, respectively, in ticks with intact legs. By contrast, the mean ESG responses with paraffin liquid (standard) in ticks with the first pair of legs surgically removed at the same concentrations were 0.039 ± 0.001, 0.026 ± 0.003, 0.026 ± 0.002, 0.011 ± 0.001, and 0.024 ± 0.002 mv, respectively.

#### 3.2.1. ESG Responses to Compounds in Ticks with Intact Legs

**Figure 3:**
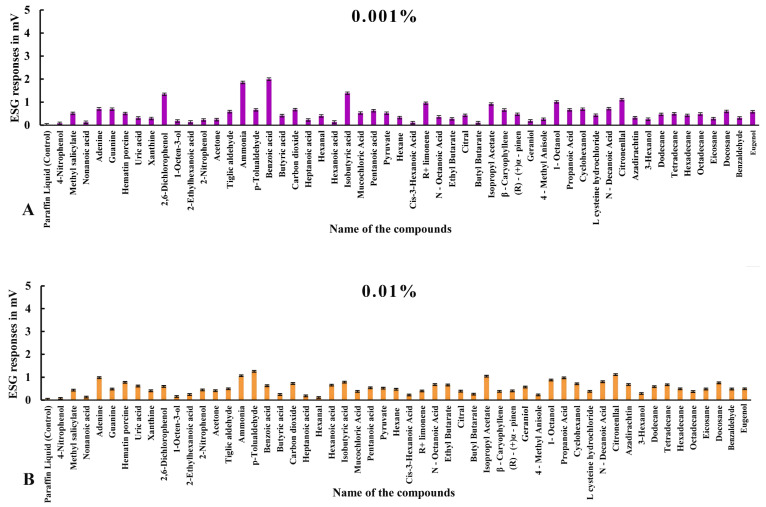
Mean normalized ESG responses in *Haemaphysalis darjeeling* with Haller’s organ to volatile organic compounds at 0.001 and 0.01% concentrations.

**Figure 4:**
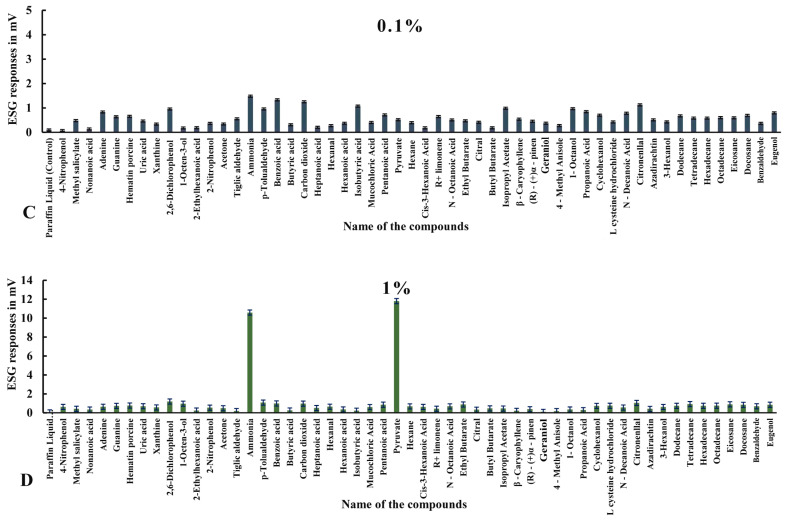
Mean normalized ESG responses in *H. darjeeling* with Haller’s organ to volatile organic compounds at 0.1 and 1% concentrations.

**Figure 5:**
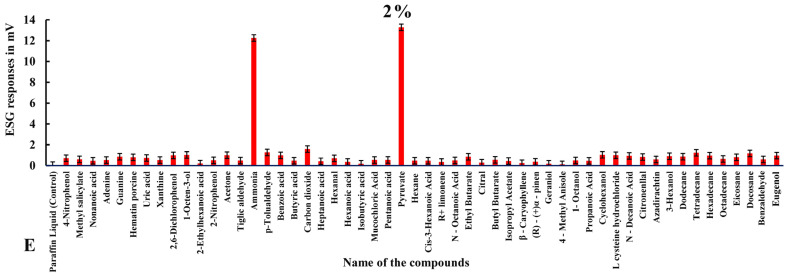
Mean normalized ESG responses in *H. darjeeling* with Haller’s organ to volatile organic compounds at 2% concentration.

Electrophysiological responses in ticks with intact legs elicited more stimulation overall (Table 2, Figure 3, Figure 4 and Figure 5). Among all the odorants, pyruvic acid showed highest ESG amplitude, recorded at a 2% concentration (13.284 ± 0.013 mv), followed by ammonia, which showed 12.256 ± 0.011 mv at 2%; benzoic acid, with 1.998 ± 0.013 mv at a concentration of 0.001%; carbon dioxide, which showed 1.589 ± 0.017 mv at a 2% concentration; isobutyric acid at 0.001% (1.385 ± 0.014 mv); 2,6-dichlorophenol, with 1.189 ± 0.013 mv at 1%; p-Tolualdehyde at a 2% concentration (1.264 ± 0.010 mv); tetradecane at 2% (1.23 ± 0.013 mv); docosane at 2% (1.17 ± 0.021 mv); citronellal at 0.1%, which evoked 1.126 ± 0.009 mv; isopropyl acetate, with 1.047 ± 0.009 mv at 0.01%; cyclohexanol, which elicited 1.031 ± 0.018 mv at a 2% concentration; 1-octane-3-ol, with 1.020 ± 0.012 mv at a 2% concentration; and 1-octanol, which showed 1.012 ± 0.015 mv at a 0.001% concentration.

Alternatively, a few other odorants showed moderate stimulation exceeding 0.5 mv, as follows: L-cysteine hydrochloride, with 0.988 ± 0.012 mv at a 2% concentration, followed by nitrogenous bases, like adenine, which elicited a maximum ESG response of 0.984 ± 0.010 mv at 0.01%; propanoic acid, which showed 0.974 ± 0.012 mv at 0.01%; eugenol, with 0.957 ± 0.008 mv at 2%; hexadecane, with 0.952 ± 0.015 mv; (R)-(+) Limonene, with 0.951 ± 0.018 mv at 0.001%; n-decanoic acid at a 2% concentration (0.927 ± 0.014 mv); 3-hexanol at 2%, showing 0.899 ± 0.014 mv; eicosane, with 0.894 ± 0.017 mv at 1%; ethyl butyrate, which responded with 0.874 ± 0.017 mv at 1%; dodecane, with 0.856 ± 0.001 mv at 2%; pentanoic acid at a 1% concentration, with 0.856 ± 0.010 mv; the nitrogenous base guanine at 2% (0.852 ± 0.014 mv); the aromatic heterocyclic compound hematin porcine, with a maximum response of 0.794 ± 0.017 mv at 2%; octadecane, with 0.748 ± 0.014 mv at 1%; uric acid at 2% (0.730 ± 0.014 mv); 4-nitrophenol, with 0.707 ± 0.016 mv at 2%; hexadecane at a 1% concentration, showing 0.698 ± 0.111 mv; hexanal, with 0.697 ± 0.013 mv at a 2% concentration; benzaldehyde at a 1% concentration, showing 0.684 ± 0.008 mv; azadirachtin, with 0.680 ± 0.038 mv at 0.01%; n-octanoic acid, with 0.680 ± 0.013 mv at 0.01%; hexane at 1%, with 0.673 ± 0.012 mv; heptadecane, with 0.659 ± 0.146 mv at 0.01%; β-caryophyllene at 0.001% (0.659 ± 0.016 mv); hexanoic acid at 0.01%, with 0.655 ± 0.013 mv; cis-3-hexanoic acid, eliciting 0.620 ± 0.014 mV at 1%; ethyl benzene, with 0.619 ± 0.181 mv at 0.01%; methyl salicylate at 2% (0.598 ± 0.015 mv); mucochloric acid, eliciting 0.598 ± 0.014 mv at a 1% concentration; tiglic aldehyde, with 0.588 ± 0.013 mv at 0.001%; tetradecane at 2% (0.574 ± 0.113 mv); geraniol, with 0.572 ± 0.012 mv stimulation at 0.01%; nimbolide at a 2% concentration, with a response of 0.566 ± 0.111 mv; xanthine at a 1% concentration (0.561 ± 0.019 mV); butyl butyrate, with 0.554 ± 0.015 mv at 2%; 2-bromo dodecane at a 0.1% concentration (0.540 ± 0.053 mv); 2-nitrophenol, with 0.536 ± 0.012 mv at 1%; and 1,2-dimethyl-propyl at 1%, with a response of 0.508 ± 0.124 mv. Other odorants with low-amplitude (<0.5 mv) responses can be seen in Figure 3, Figure 4 and Figure 5 and Table 2 in specimens with intact legs.

#### 3.2.2. ESG Responses to Compounds in Ticks with Surgically Removed Legs

ESG responses to the aforesaid odorants in ticks with the first pair of legs surgically removed showed a similar intriguing pattern of variability in ESG peaks, but generally, the stimulations were of low amplitude (Figures S2–S4, Table 3).

### 3.3. One-Way ANOVA and Post Hoc Tukey Test in Ticks with Intact Legs

To investigate the average differences among the five groups (concentrations of *w*/*v* 0.001%, 0.01%, 0.1%, 1%, and 2%), one-way ANOVA was performed on intact ticks. Table S1 displays the corresponding mean differences and variances that were computed. It was discovered through this multiple comparison of the ESG amplitude responses at various doses that distinct groups could be placed into homogeneous subsets. Within homogeneous groups, no discernible variations were seen. As shown in Table S1A, at 0.001, 0.01, and 0.1% *w*/*v* concentrations, the homogeneous groups showed mean values of 0.542, 0.541, and 0.577, respectively, while at *w*/*v* 1 and 2% concentrations, the homogeneous set showed mean values of 1.006 and 1.124, respectively. Observing the one-way ANOVA (Table S1B), the alpha is smaller than *p* at a 0.05 significance level, which indicates that the null hypothesis is true and that the means of the various concentrations do not differ significantly. As confirmation, a post hoc Turkey HSD was also performed for the ANOVA results, and the data are presented in Table S1C. However, no significant differences were found among the groups.

#### 3.3.1. One-Way ANOVA Between Different Conditions

Furthermore, to show changes in response between two groups (ticks with intact legs and those with Haller’s organ surgically removed), one-way ANOVA across samples at five different concentrations was performed (Table S1D). In the samples at lower concentrations, such as 0.001%, 0.01%, and 0.1% *w*/*v*, the Type 1 error values were 7.2%, 1.1%, and 2.2%, respectively. At high concentrations of 1% and 2%, the test showed a probability of Type 1 error of 0.007%. Since the *p*-value is <α, H_o_ is rejected again, indicating that the sample difference between the average of some groups is large enough to be statistically significant. From the results, it can be predicted that the presence of Haller’s organ makes a significant difference in response across all concentrations. However, at a 1% concentration, it showed the effect with the lowest probability of Type 1 error or *p*-value.

#### 3.3.2. Determination of R-Squared Regression Values

The investigation identified and analyzed *R*-squared values, which are regression models’ goodness-of-fit measurements (https://doi.org/10.5281/zenodo.14849790 (accessed on 5 May 2025)). The fundamental theorem of algebra states that there are three solutions to the equation since it is a third-order polynomial. The gradient of a quadratic function varies from point to point. It was found by evaluating the function at the relevant point and computing its derivative. Among 55 VOCs, twenty compounds—*viz*. hematin porcine(R)-(+) Limonene, 2-nitrophenol, cis-3-hexanoic acid, carbon dioxide, uric acid, 1-octanol, butyric acid, methyl salicylate, ethyl butyrate, xanthine, azadirachtin, β-caryophyllene, benzoic acid, (R)-(+)α-pinene, benzaldehyde, tiglic aldehyde, 2-ethylhexanoic acid, n-octanoic acid, hexane, and paraffin liquid (control)—showed R^2^ values between 0.5 and 0.8, indicating moderately responsive compounds. Other volatile compounds, like hexadecane, geraniol, dodecane, hexanal, pentanoic acid, adenine, cyclohexanol, acetone, octadecane, docosane, L-cysteine hydrochloride, tetradecane, nonanoic acid, eicosane, 2,6-dichlorophenol, citronellal, guanine, ammonia, isopropyl acetate, mucochloric acid, propanoic acid, 4-nitrophenol, 1-octen-3-ol, eugenol, pyruvate, citral, heptanoic acid, butyl butyrate, *p*-tolualdehyde, n-decanoic acid, 4-methyl anisole, hexanoic acid, and isobutyric acid, showed R-squared values between 1 and 0.8, indicating the most responsive compounds.

### 3.4. Olfactometric Bioassays

#### 3.4.1. Multi-Arm Olfactometric Assay

**Figure 6:**
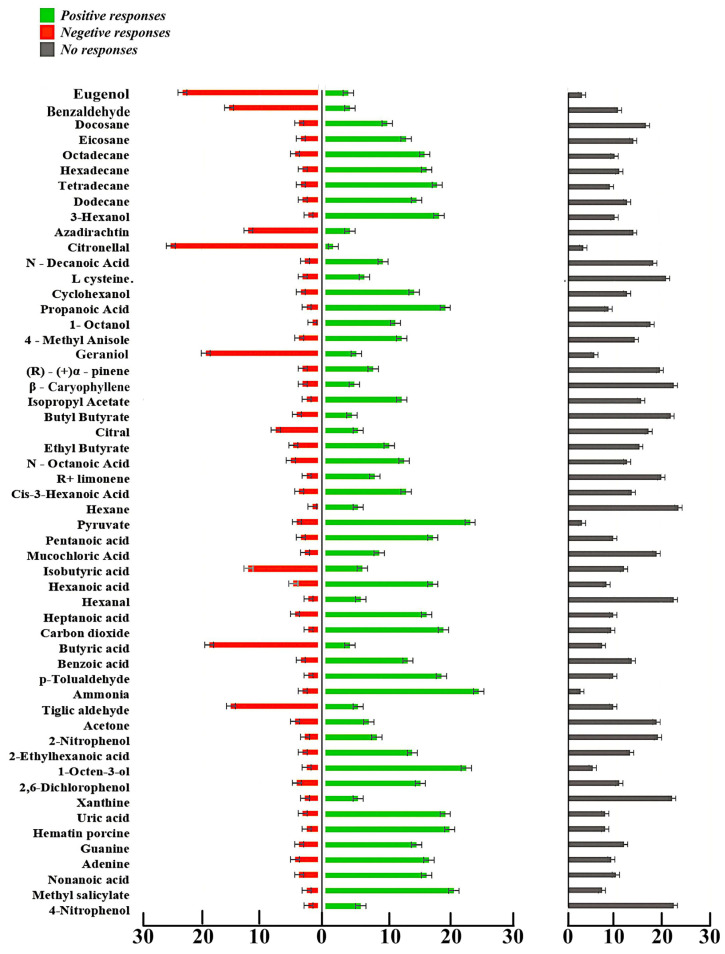
Multichannel bioassay response of *Haemaphysalis darjeeling* at *w*/*v* 2% concentration. A total of three replicates with 30 individuals each were studied in this assay (N = 90). (Total mean ESG response was highest in the group at 2% concentration; hence, this concentration was chosen for olfactometric assay).

Figure 6 shows a bar graph depiction of the eight-arm olfactometric analysis in *H. darjeeling*. The experiment was carried out in accordance with the experimental procedure described in the materials and methods section. Table 4 shows the number of ticks that moved to the arm of the olfactometer in the eight-arm olfactometric bioassay, along with the respective binomial test analysis. Based on the values listed in Table 3, the compounds that showed best positive responses in more than 50% of ticks were ammonia (82.3%), pyruvate (77.6%), 1-octen-3-ol (75.5%), methyl salicylate (69%), hematin porcine (66.6%), uric acid (64%), propanoic acid (64%), carbon dioxide (63.33%), p-Tolualdehyde (62.33%), 3-hexanol (61%), Tetradecane (18, 60%), pentanoic acid (57%), hexanoic acid (56.66%), tetradecane (56.03%), adenine (56.00%), heptanoic acid (54%), hexadecane (54%), nonanoic acid (54.00%), octadecane (53%), and 2,6-dichlorophenol (51%). Compounds with strong negative responses toward the same species were citronellal (84%), eugenol (77%), butyric acid (64%), geraniol (62%), benzaldehyde (51%), and tiglic aldehyde (50%). On the other hand, with paraffin liquid, which was used as a solvent, the ticks did not show positive or negative responses, remaining almost non-responsive in all trials. The result for positive responses was 1 ± 0.6, the result for negative responses was 0.3 ± 0.3, and for the no-response category, it was 28.7 ± 0.9, with chi-square and *p*-values of 52.28 and >0.05, indicating that the behavior of the specimens remained unaltered by paraffin liquid.

The categorization of the compounds was further supported by binomial analysis. Based on the binomial test, in each case with *p*-values at the 0.05 level of significance that were lower than the expected values, the category (positive or negative) that showed the highest count of ticks was considered the final response. For the compounds 4-nitrophenol, xanthine, 2-nitrophenol, acetone, hexanal, isobutyric acid, mucochloric acid, hexane, (R)-(+) Limonene, n-octanoic acid, ethyl butyrate, citral, butyl butyrate, β-caryophyllene, (R)-(+)α-pinene, and L-cysteine hydrochloride. The binomial test showed higher *p*-values, with no significant differences between negative and positive responses. Simultaneously, for each of the above compounds, the ‘no response’ category carried a much higher count of ticks than the positive and negative groups, which indicated that the compounds had the lowest probability of showing behavioral changes. However, among the significant response groups, i.e., tiglic aldehyde, butyric acid, geraniol, citronellal, benzaldehyde, and eugenol, ticks showed higher counts for the negative response category, while with the rest of the compounds, ticks showed higher counts for positive responses.

#### 3.4.2. Y-Tube Olfactometric Assay

**Figure 7:**
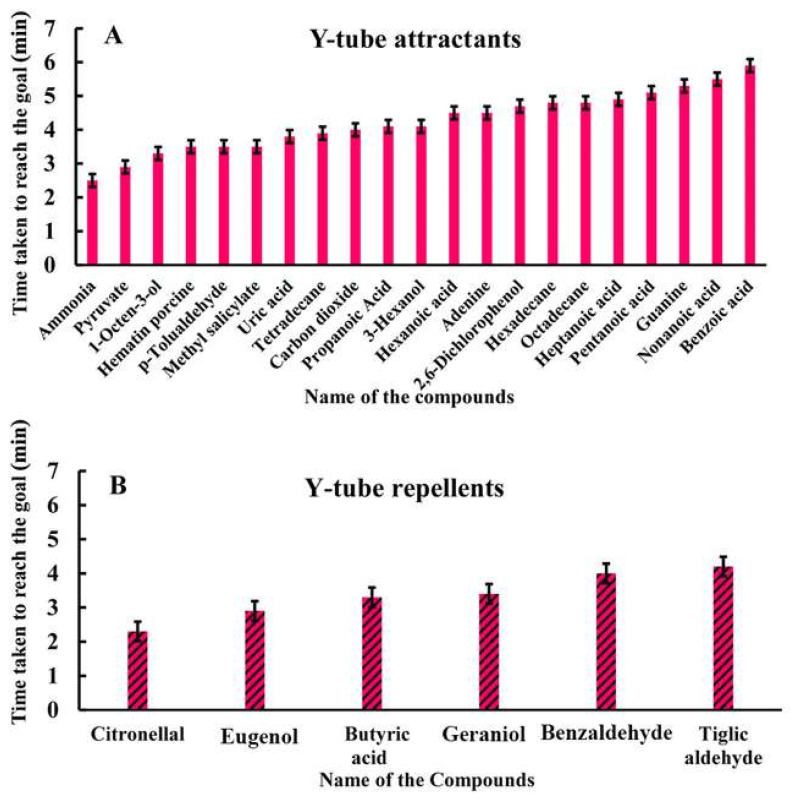
Y-tube bioassay response of selected compounds showing strong (**A**) positive and (**B**) negative responses in more than 50% of ticks in eight-arm olfactometer in *Haemaphysalis darjeeling* at *w*/*v* 2% concentration.

Following the above behavioral bioassay, only compounds that showed strong positive and negative responses in more than 50% of ticks in the eight-arm olfactometer were further studied using Y-tube analysis (Figure 7). The experiment was conducted following the experimental protocol described in the materials and methods section for the Y-tube bioassay. According to the assay, the ticks were observed to take the least time to reach the Y-tube arm laced with ammonia (2.5 min), followed by pyruvate (2.9 min), 1-octen-3-ol (3.3 min), hematin porcine (3.4 min), p-Tolualdehyde (3.5 min), methyl salicylate (3.5 min), uric acid (3.8 min), tetradecane (3.9 min), carbon dioxide (4.0 min), propanoic acid (4.1 min), 3-hexanal (4.4 min), hexanoic acid (4.5 min), adenine (4.5 min), 2,6-dichlorophenol (4.7 min), hexadecane (4.8 min), heptanoic acid (4.9 min), pentanoic acid (5.1 min), octadecane (5.3 min), guanine (5.3 min), and nonanoic acid (5.5 min) (Table 5). This trend showed that ticks moved toward the arm laced with VOCs, indicating a decreasing favorable or attracted behavior toward those compounds. However, the ticks were observed to take the shortest time to reach the vacant arm of the Y-tube when the other arm was laced with volatile organic compounds—*viz.* citronellal (2.3 min), followed by eugenol (2.9 min), (3.9 min), butyric acid (3.3 min), geraniol (3.4 min), benzaldehyde (4.0 min), and tiglic aldehyde (4.2 min), showing descending repellant activities of the VOCs (Table 5).

## 4. Discussion

We showed the first evidence ofthe detailed structure and function of Haller’s organ for chemosensation in *Haemaphysalis darjeeling*, which acts as an exceptional biosensor for monitoring and analyzing chemical cues. The sensory organs of ticks are different from typical insects’ olfactory systems [12,53,55,56,57]. Olfactory sensilla in ticks are believed to be present mostly in the paired Haller’s structure, which is found on the first tarsal segment of the forelegs [12,20,21], while insect antennae are paired appendages attached to the head, typically consisting of a basal scape, pedicel, and a flagellum with multiple segments, where sensilla are distributed along the entire length, often with specialized zones for different sensory functions [53,56,58]. Haller’s organ is primarily specialized for detecting host odors, CO_2_, humidity, and temperature changes. Insect antennae, on the other hand, have more diverse functionality, including olfaction, gustation, mechanoreception, thermoreception, hygroreception, and sometimes communication [52,56,59]. Haller’s organ is formed of neuronal projections and olfactory glomeruli [40]. Although no sexual dimorphism was observed in this investigation in terms of the structure and distribution of sensilla of Haller’s structure in *H. darjeeling*, ticks are generally observed to exhibit diversity in anterior pit sensilla [45,46]. Moreover, Haller’s capsule with slits is highly distinct and appears to be species-specific. Such distinctions may be employed in the biosystematics of ticks [45,60]. The organization, form, and number of anterior pit sensilla play a crucial role in recognizing many species of ticks [45,60]. The secretion of multicellular glands (as documented in the microscopy study) underlying the capsule may be involved in the diffusion of odor molecules activating the sensilla’s dendritic membrane. The capsule is thought to act primarily as a protective device to protect the thin-walled and non-socketed capsule sensilla. The microscopy investigation of Haller’s organ further revealed the striated muscles that act as a specific anchor for the cuticle. Phylogenetically, Haller’s structures evolved in ticks (Ixodida), which are arachnids belonging to the subphylum Chelicerata, whereas insect antennae are derived from ancestral appendages in the mandibulate arthropod lineage, belonging to subphylum Mandibulata. Regarding adaptation, Haller’s organ is highly specialized for the parasitic lifestyle of ticks, particularly for locating hosts, whereas insect antennae evolved for more generalized environmental sensing, with subsequent specialization for diverse ecological niches. Haller’s structure is thus not homologous to insect antennae. It represents the independent evolution of a sensory structure on a walking appendage, while insect antennae are homologous across all insect groups, derived from ancestral head appendages [9,59,61,62]. These fundamental differences reflect distinct evolutionary paths of chelicerates (including ticks) and mandibulates (including insects), which diverged over 550 million years ago in the early Cambrian period.

Furthermore, the results from electrophysiological and olfactometric bioassays unequivocally revealed the chemoreceptive role of Haller’s organ in ticks. The synganglion (Figure S1) plays an important role in processing ESG responses and represents the tick’s central nervous system, where all chemosensory information is analyzed [20,28,63]. After removing the first pair of legs, which house chemosensory structures related to Haller’s organ, ESG showed much weaker stimulation compared to the response in ticks with intact legs, with a significant difference (*p* < 0.001). However, the stimulation was not affected (*p* < 0.05) by the surgical amputation of the second pair of appendages. Apart from this, it has also been reported by other investigators that chemical cues are even detected by multimodal sensilla on mouthparts [11,20,28]. Besides Haller’s organ, palpal sensilla, tarsal sensilla, genual organ, and various other chemoreceptors distributed across the body surface of ticks may partially compensate for specific functions [11,20,28,64]. Statistical analysis further showed no significant differences among the five different concentrations in tick specimens with intact legs. By contrast, one-way ANOVA showed a significant difference between the two conditions (ticks with intact legs and those with Haller’s organ removed) at five different concentrations. Furthermore, *R*^2^ regression values of the VOCs at different doses close to 1 showed the most responsive compounds, while values between 0.8 and 0.5 showed moderately responsive compounds.

Together with the electrophysiological analysis, ticks were tested on different behavioral assays. Behavioral tests that evaluate specimens’ attraction/repulsion to specific odors are inherently laborious, time-consuming, and require several replications. Multichannel olfactometer testing is performed using a number of odor sources simultaneously. In the present study, an eight-arm olfactometer was utilized to determine whether certain organic compounds were attractants or repellents to ticks. Four chemicals were applied at a time to replicate a natural environment with a multitude of compounds for the specimen to identify, while four additional arms were left unoccupied. Following the multichannel bioassay, the VOCs that elicited strong negative (repellent) or positive (attractant) responses were selected as single compounds for the Y-tube analysis. This allowed the tick to choose between the two arms, and the time it took to move toward the chemically treated or unoccupied arm, which depended on the nature of the odorants, was measured. As a result, the two behavioral assays provided data on the number of specimens, along with the time spent by the ticks moving toward the arms, allowing us to determine whether the VOCs were powerful repellents or attractants. Based on these two behavioral bioassays, ammonia, followed by pyruvate, 1-octane-3-ol, hematin porcine, p-Tolualdehyde, methyl salicylate, uric acid, tetradecane, carbon dioxide, propanoic acid, 3-hexanol, hexanoic acid, adenine, 2,6-dichlorophenol, hexadecane, heptanoic and pentanoic acid, octadecane, guanine, and nonanoic acid were the shortlisted compounds have been categorized as tick attractants. Most of these shortlisted attractant compounds are reported to be common host kairomones and pheromonal components [21,23,27,28,49,53]. The study also showed tick behavioral reactions of increased attraction toward short-chain carboxylic compounds, indicating a link between molecular structure and behavior in the species [28,65]. Moreover, straight-chain saturated hydrocarbons, or n-alkanes (C12, C14, C16, C18, C20, and C22), showed tick attractant properties in the olfactometric assay. Straight-chain saturated hydrocarbons, or n-alkanes, are common constituents of plant volatiles [53,65,66]. However, volatile chemical compounds, such as citronellal, eugenol, butyric acid, geraniol, benzaldehyde, and tiglic aldehyde, showed a decreasing repellent tendency in the same species, as analyzed by olfactometric bioassays. Citronellal is a popular plant-based mosquito repellent, which has been shown to work against *Drosophila melanogaster* through olfactory pathways [67]. Recently, eugenol and benzaldehyde have been reported to show effective in vitro acaricidal activity against *Rhipicephalus annulatus* [68]. Geraniol, a monoterpene alcohol, was found to be effective against *Psoroptes ovis* both in vitro and in vivo [69], and it was shown to be an effective insect repellent [70]. Several studies have investigated the chemical structure and the significance of various moieties in the attractant and repellent qualities of tick olfaction [50,55,63,71]. In any event, the findings of this experiment reveal the influence of molecular structure on the bioactivity of attractant and repellent compounds in ticks, as has previously been discovered in other arthropods and vertebrates [23,54,56,57,71]. In general, odorants are detected by numerous olfactory receptors with varying affinities, and each receptor has an odor response profile [72,73,74,75]. Few references related to the suppression of cholinesterase and cytochrome P450s in ticks by repellents have reported the most likely deterrent mechanism in ticks [41,76]. Furthermore, repellents have been observed to abolish thermotaxis without influencing olfaction-stimulated host-seeking behaviors [43]. However, more research into the role of steric and chemical interactions between the compounds and receptors in chemosensory systems is needed. Additionally, bioinformatic prediction of G-protein-coupled receptors (GPCRs) using a foreleg transcriptome of ticks has revealed that the GPCR pathway is also implicated in tick olfaction [40,77].

Even though these natural VOCs have shown great promise in controlled laboratory settings, their effectiveness against tick populations in actual field conditions is frequently diminished. Laboratory studies typically test VOCs in sealed chambers with constant temperature and humidity, whereas field conditions involve fluctuating temperatures, precipitation, and wind and air currents that can rapidly dissipate volatile compounds. Furthermore, the high volatility that makes VOCs effective in confined spaces causes rapid dissipation outdoors, requiring frequent reapplication. UV radiation and oxidation in field settings further accelerate the degradation of these plant compounds, significantly reducing their effective lifespan. However, suitable formulations in correct proportions or microencapsulations are expected to solve these problems [78].

## 5. Conclusions

In summary, ticks with Haller’s organs demonstrated high stimulation in response to various organic compounds. However, when Haller’s organ was removed, almost no olfactory response was detected. Thus, Haller’s structure, a biosensor, might be a target for olfaction-based tick control research in the future. Based on the above investigation, citronellal, followed by eugenol, butyric acid, geraniol, benzaldehyde, and tiglic aldehyde, are the compounds shortlisted as strong tick repellents. Future research will focus on designing and formulating effective repellent chemicals to control ticks, including their mechanism of action and targeted pathways in both laboratory and field conditions. More replications with different VOCs and concentration ranges are required to better understand the repellent dose–response connection in tick behavioral responses.

## Figures and Tables

**Table 1:** List of the organic compounds used in electroscutumography (ESG) and olfactometry for the Ixodidae tick *Haemaphysalis darjeeling*.

Sl No.	Name of the Compound	Purity	Supplier
1	4-Nitrophenol	≥99%	Sigma
2	Methyl salicylate	≥99%	Sigma Aldrich
3	Nonanoic acid	≥97%	Sigma Aldrich
4	Adenine	≥99%	Merck
5	Guanine	≥98%	Merck
6	Hematin porcine	-	Sigma Aldrich
7	Uric acid	≥99%	Sigma Aldrich
8	Xanthine	≥99%	Sigma
9	2,6-Dichlorophenol	≥99%	Sigma Aldrich
10	1-Octen-3-ol	≥98%	Aldrich
11	2-Ethylhexanoic acid	-	Aldrich
12	2-Nitrophenol	≥98%	Aldrich
13	Acetone	≥99.5%	Sigma-Aldrich
14	Tiglic aldehyde	≥96%	Aldrich
15	Ammonia	≥25%	CDH
16	p-Tolualdehyde	≥97%	Sigma-Aldrich
17	Benzoic acid	≥99.5%	Sigma-Aldrich
18	Butyric acid	≥99%	Sigma-Aldrich
19	Carbon dioxide	≥99.8%	Sigma-Aldrich
20	Heptanoic acid	≥99%	Sigma-Aldrich
21	Hexanal	≥98%	Aldrich
22	Hexanoic acid	≥99%	Sigma-Aldrich
23	Isobutyric acid	≥99%	Sigma-Aldrich
24	Mucochloric acid	≥99%	Sigma-Aldrich
25	Pentanoic acid	≥99%	Sigma-Aldrich
26	Pyruvate	≥99%	Sigma-Aldrich
27	Hexane	≥95%	Sigma
28	cis-3-Hexanoic acid	-	Sigma-Aldrich
29	(R)-(+) Limonene	≥97%	Sigma-Aldrich
30	n-Octanoic acid	≥97.5%	CDH
31	Ethyl butyrate	≥99%	Sigma-Aldrich
32	Citral	≥95%	Sigma-Aldrich
33	Butyl butyrate	≥98%	Sigma-Aldrich
34	Isopropyl acetate	≥99.6%	Sigma-Aldrich
35	β-Caryophyllene	≥80%	Sigma-Aldrich
36	(R)-(+)α-pinene	-	Sigma-Aldrich
37	Geraniol	≥98%	Sigma-Aldrich
38	4-Methyl anisole	≥99%	Sigma-Aldrich
39	1-Octanol	≥99%	CDH
40	Propanoic acid	≥99.5%	Sigma-Aldrich
41	Cyclohexanol	≥99%	Sigma-Aldrich
42	L-Cysteine hydrochloride	-	Sigma-Aldrich
43	n-Decanoic acid	≥98%	Sigma-Aldrich
44	Citronellal	≥98%	CDH
45	Azadirachtin	≥95%	Sigma-Aldrich
46	3-Hexanol	≥97%	Sigma-Aldrich
47	Dodecane	≥99%	Merck
48	Tetradecane	≥99%	Sigma-Aldrich
49	Hexadecane	≥99%	Sigma-Aldrich
50	Octadecane	≥99%	Sigma-Aldrich
51	Eicosane	≥99%	Sigma-Aldrich
52	Docosane	≥99%	Sigma-Aldrich
53	Benzaldehyde	≥99.5%	Sigma-Aldrich
54	Eugenol	≥99%	Sigma-Aldrich
55	Paraffin liquid light (control, used as solvent)	≥30%	Merck

**Table 2:** Electroscutumography responses of Ixodidae tick *Haemaphysalis darjeeling* with intact legs (all responses recorded in mv).

SL No.	Name of the Compound	Concentration (*w*/*v*)				
		0.001%	0.01%	0.1%	1%	2%
1	4-Nitrophenol	0.082 ± 0.014	0.071 ± 0.009	0.074 ± 0.009	0.619 ± 0.015	0.707 ± 0.016
2	Methyl salicylate	0.512 ± 0.014	0.433 ± 0.017	0.480 ± 0.01	0.416 ± 0.01	0.598 ± 0.015
3	Nonanoic acid	0.129 ± 0.015	0.131 ± 0.011	0.137 ± 0.01	0.334 ± 0.017	0.463 ± 0.011
4	Adenine	0.705 ± 0.015	0.984 ± 0.010	0.833 ± 0.010	0.636 ± 0.016	0.531 ± 0.015
5	Guanine	0.697 ± 0.014	0.483 ± 0.015	0.637 ± 0.015	0.725 ± 0.017	0.852 ± 0.014
6	Hematin porcine	0.509 ± 0.014	0.779 ± 0.014	0.655 ± 0.011	0.761 ± 0.011	0.794 ± 0.017
7	Uric acid	0.311 ± 0.012	0.616 ± 0.013	0.463 ± 0.013	0.688 ± 0.018	0.730 ± 0.014
8	Xanthine	0.290 ± 0.016	0.408 ± 0.014	0.337 ± 0.013	0.561 ± 0.019	0.530 ± 0.013
9	2,6-Dichlorophenol	1.340 ± 0.015	0.600 ± 0.015	0.956 ± 0.011	1.189 ± 0.013	0.977 ± 0.015
10	1-Octen-3-ol	0.180 ± 0.014	0.157 ± 0.017	0.179 ± 0.012	0.958 ± 0.012	1.020 ± 0.012
11	2-Ethylhexanoic acid	0.139 ± 0.008	0.244 ± 0.016	0.189 ± 0.009	0.235 ± 0.014	0.191 ± 0.012
12	2-Nitrophenol	0.228 ± 0.014	0.444 ± 0.014	0.367 ± 0.012	0.536 ± 0.012	0.505 ± 0.016
13	Acetone	0.240 ± 0.013	0.412 ± 0.014	0.340 ± 0.013	0.477 ± 0.013	0.995 ± 0.01
14	Tiglic aldehyde	0.588 ± 0.013	0.495 ± 0.016	0.553 ± 0.009	0.174 ± 0.014	0.479 ± 0.014
15	Ammonia	1.854 ± 0.017	1.066 ± 0.014	1.481 ± 0.01	10.585 ± 0.018	12.256 ± 0.011
16	*p*-Tolualdehyde	0.666 ± 0.013	1.261 ± 0.015	0.958 ± 0.012	1.072 ± 0.015	1.264 ± 0.010
17	Benzoic acid	1.998 ± 0.013	0.630 ± 0.014	1.331 ± 0.07	0.986 ± 0.013	0.980 ± 0.012
18	Butyric acid	0.415 ± 0.013	0.245 ± 0.013	0.310 ± 0.011	0.240 ± 0.010	0.467 ± 0.012
19	Carbon dioxide	0.675 ± 0.013	0.726 ± 0.016	1.252 ± 0.010	0.966 ± 0.013	1.589 ± 0.017
20	Heptanoic acid	0.228 ± 0.018	0.182 ± 0.014	0.205 ± 0.013	0.501 ± 0.015	0.406 ± 0.013
21	Hexanal	0.401 ± 0.016	0.112 ± 0.011	0.273 ± 0.09	0.639 ± 0.012	0.697 ± 0.013
22	Hexanoic acid	0.137 ± 0.018	0.655 ± 0.013	0.371 ± 0.013	0.350 ± 0.016	0.351 ± 0.015
23	Isobutyric acid	1.385 ± 0.014	0.785 ± 0.015	1.074 ± 0.016	0.214 ± 0.017	0.175 ± 0.010
24	Mucochloric acid	0.526 ± 0.015	0.381 ± 0.012	0.403 ± 0.010	0.598 ± 0.014	0.537 ± 0.013
25	Pentanoic acid	0.626 ± 0.013	0.538 ± 0.012	0.714 ± 0.013	0.856 ± 0.010	0.544 ± 0.012
26	Pyruvate	0.520 ± 0.017	0.524 ± 0.012	0.517 ± 0.008	11.796 ± 0.011	13.284 ± 0.013
27	Hexane	0.333 ± 0.008	0.474 ± 0.013	0.394 ± 0.012	0.673 ± 0.012	0.466 ± 0.012
28	cis-3-Hexanoic acid	0.110 ± 0.013	0.223 ± 0.015	0.184 ± 0.013	0.620 ± 0.014	0.466 ± 0.009
29	(R)-(+) Limonene	0.951 ± 0.018	0.399 ± 0.013	0.646 ± 0.012	0.428 ± 0.016	0.345 ± 0.009
30	n-Octanoic Acid	0.360 ± 0.015	0.680 ± 0.013	0.507 ± 0.013	0.677 ± 0.009	0.496 ± 0.015
31	Ethyl butyrate	0.275 ± 0.017	0.661 ± 0.016	0.472 ± 0.012	0.874 ± 0.017	0.849 ± 0.015
32	Citral	0.428 ± 0.014	0.392 ± 0.014	0.413 ± 0.010	0.322 ± 0.015	0.281 ± 0.009
33	Butyl butyrate	0.111 ± 0.014	0.260 ± 0.012	0.185 ± 0.010	0.462 ± 0.013	0.554 ± 0.015
34	Isopropyl acetate	0.918 ± 0.014	1.047 ± 0.009	0.987 ± 0.014	0.444 ± 0.018	0.438 ± 0.013
35	β-Caryophyllene	0.659 ± 0.016	0.382 ± 0.014	0.539 ± 0.012	0.203 ± 0.014	0.227 ± 0.014
36	(R)-(+)α-pinene	0.472 ± 0.015	0.402 ± 0.012	0.452 ± 0.015	0.367 ± 0.009	0.365 ± 0.012
37	Geraniol	0.183 ± 0.015	0.572 ± 0.012	0.373 ± 0.014	0.890 ± 0.011	0.776 ± 0.013
38	4-Methyl anisole	0.256 ± 0.008	0.225 ± 0.012	0.278 ± 0.014	0.172 ± 0.019	0.118 ± 0.017
39	1-Octanol	1.012 ± 0.015	0.880 ± 0.011	0.967 ± 0.008	0.342 ± 0.011	0.492 ± 0.014
40	Propanoic acid	0.667 ± 0.014	0.974 ± 0.012	0.847 ± 0.012	0.277 ± 0.013	0.456 ± 0.015
41	Cyclohexanol	0.693 ± 0.011	0.714 ± 0.016	0.702 ± 0.012	0.721 ± 0.011	1.031 ± 0.018
42	L-cysteine hydrochloride	0.431 ± 0.014	0.382 ± 0.016	0.422 ± 0.011	0.741 ± 0.022	0.988 ± 0.012
43	n-Decanoic acid	0.715 ± 0.012	0.805 ± 0.017	0.781 ± 0.012	0.554 ± 0.021	0.927 ± 0.014
44	Citronellal	1.106 ± 0.011	1.117 ± 0.014	1.126 ± 0.009	1.04 ± 0.011	0.823 ± 0.013
45	Azadirachtin	0.322 ± 0.047	0.680 ± 0.038	0.510 ± 0.062	0.405 ± 0.042	0.589 ± 0.127
46	3-Hexanol	0.259 ± 0.012	0.288 ± 0.015	0.426 ± 0.012	0.606 ± 0.014	0.899 ± 0.014
47	Dodecane	0.467 ± 0.014	0.589 ± 0.010	0.674 ± 0.010	0.737 ± 0.014	0.856 ± 0.001
48	Tetradecane	0.494 ± 0.017	0.672 ± 0.013	0.584 ± 0.014	0.916 ± 0.016	1.23 ± 0.013
49	Hexadecane	0.426 ± 0.018	0.491 ± 014	0.583 ± 0.014	0.738 ± 0.010	0.952 ± 0.015
50	Octadecane	0.485 ± 0.014	0.372 ± 0.008	0.603 ± 0.013	0.748 ± 0.014	0.637 ± 0.014
51	Eicosane	0.283 ± 0.016	0.485 ± 0.017	0.596 ± 0.014	0.894 ± 0.017	0.799 ± 0.02
52	Docosane	0.596 ± 0.013	0.754 ± 0.013	0.695 ± 0.011	0.834 ± 0.014	1.17 ± 0.021
53	Benzaldehyde	0.315 ± 0.010	0.485 ± 0.017	0.369 ± 0.017	0.684 ± 0.008	0.593 ± 0.013
54	Eugenol	0.583 ± 0.021	0.495 ± 0.013	0.795 ± 0.010	0.848 ± 0.010	0.957 ± 0.008

**Table 3:** Electroscutumography (ESG) responses of Ixodidae ticks *Haemaphysalis darjeeling* after the removal of first pair appendages (all responses recorded in mv).

SL No.	Name of the Compound	Concentration (*w*/*v*)				
		0.001%	0.01%	0.1%	1%	2%
1	4-Nitrophenol	0.024 ± 0.001	0.022 ± 0.002	0.196 ± 0.006	0.168 ± 0.009	0.156 ± 0.002
2	Methyl salicylate	0.120 ± 0.001	0.060 ± 0.009	0.117 ± 0.005	0.069 ± 0.014	0.062 ± 0.003
3	Nonanoic acid	0.029 ± 0.001	0.043 ± 0.003	0.114 ± 0.006	0.050 ± 0.001	0.150 ± 0.002
4	Adenine	0.172 ± 0.001	0.122 ± 0.003	0.145 ± 0.007	0.099 ± 0.013	0.126 ± 0.001
5	Guanine	0.035 ± 0.003	0.274 ± 0.007	0.153 ± 0.004	0.263 ± 0.002	0.089 ± 0.001
6	Hematin porcine	0.126 ± 0.001	0.221 ± 0.007	0.189 ± 0.007	0.098 ± 0.013	0.187 ± 0.001
7	Uric acid	0.045 ± 0.001	0.141 ± 0.003	0.218 ± 0.005	0.078 ± 0.008	0.115 ± 0.002
8	Xanthine	0.036 ± 0.001	0.052 ± 0.003	0.125 ± 0.006	0.304 ± 0.004	0.298 ± 0.001
9	2,6-Dichlorophenol	0.213 ± 0.001	0.086 ± 0.006	0.322 ± 0.008	0.254 ± 0.007	0.210 ± 0.001
10	1-Octen-3-ol	0.031 ± 0.009	0.055 ± 0.008	0.054 ± 0.005	0.210 ± 0.009	0.285 ± 0.002
11	2-Ethylhexanoic acid	0.028 ± 0.001	0.045 ± 0.007	0.040 ± 0.002	0.022 ± 0.004	0.043 ± 0.001
12	2-Nitrophenol	0.030 ± 0.001	0.113 ± 0.004	0.333 ± 0.009	0.122 ± 0.005	0.147 ± 0.002
13	Acetone	0.094 ± 0.001	0.105 ± 0.003	0.227 ± 0.002	0.281 ± 0.010	0.093 ± 0.001
14	Tiglic aldehyde	0.066 ± 0.001	0.070 ± 0.009	0.071 ± 0.009	0.069 ± 0.009	0.045 ± 0.002
15	Ammonia	0.687 ± 0.001	0.200 ± 0.002	0.392 ± 0.005	4.516 ± 0.013	4.199 ± 0.002
16	*p*-Tolualdehyde	0.110 ± 0.001	0.240 ± 0.007	0.324 ± 0.005	0.252 ± 0.012	0.276 ± 0.002
17	Benzoic acid	0.569 ± 0.001	0.095 ± 0.008	0.205 ± 0.006	0.208 ± 0.001	0.196 ± 0.002
18	Butyric acid	0.094 ± 0.001	0.052 ± 0.004	0.069 ± 0.007	0.055 ± 0.007	0.027 ± 0.002
19	Carbon dioxide	0.426 ± 0.008	0.544 ± 0.013	0.275 ± 0.018	0.174 ± 0.006	0.363 ± 0.003
20	Heptanoic acid	0.034 ± 0.000	0.066 ± 0.009	0.039 ± 0.003	0.063 ± 0.008	0.107 ± 0.002
21	Hexanal	0.075 ± 0.009	0.056 ± 0.004	0.118 ± 0.005	0.101 ± 0.006	0.140 ± 0.002
22	Hexanoic acid	0.047 ± 0.001	0.063 ± 0.004	0.041 ± 0.001	0.073 ± 0.013	0.114 ± 0.002
23	Isobutyric acid	0.269 ± 0.0008	0.135 ± 0.01	0.104 ± 0.009	0.055 ± 0.004	0.048 ± 0.002
24	Mucochloric acid	0.066 ± 0.001	0.287 ± 0.003	0.068 ± 0.006	0.121 ± 0.014	0.074 ± 0.002
25	Pentanoic acid	0.035 ± 0.005	0.157 ± 0.006	0.245 ± 0.025	0.042 ± 0.014	0.137 ± 0.003
26	Pyruvate	0.057 ± 0.001	0.050 ± 0.004	0.179 ± 0.008	0.469 ± 0.010	0.733 ± 0.003
27	Hexane	0.053 ± 0.001	0.071 ± 0.003	0.113 ± 0.002	0.104 ± 0.010	0.195 ± 0.001
28	cis-3-Hexanoic acid	0.046 ± 0.0009	0.049 ± 0.002	0.065 ± 0.006	0.127 ± 0.012	0.139 ± 0.002
29	(R)-(+) Limonene	0.174 ± 0.001	0.197 ± 0.006	0.115 ± 0.008	0.062 ± 0.008	0.057 ± 0.002
30	N-Octanoic acid	0.056 ± 0.001	0.151 ± 0.004	0.060 ± 0.004	0.088 ± 0.008	0.063 ± 0.002
31	Ethyl butyrate	0.049 ± 0.001	0.132 ± 0.002	0.091 ± 0.009	0.205 ± 0.009	0.130 ± 0.002
32	Citral	0.126 ± 0.001	0.067 ± 0.005	0.269 ± 0004	0.110 ± 0.007	0.067 ± 0.002
33	Butyl butyrate	0.032 ± 0.001	0.082 ± 0.007	0.063 ± 0.004	0.072 ± 0.004	0.060 ± 0.001
34	Isopropyl acetate	0.237 ± 0.001	0.186 ± 0.003	0.064 ± 0.008	0.040 ± 0.005	0.042 ± 0.002
35	β-Caryophyllene	0.161 ± 0.001	0.070 ± 0.004	0.066 ± 0.002	0.081 ± 0.010	0.047 ± 0.002
36	(R)-(+)α-pinene	0.063 ± 0.001	0.108 ± 0.002	0.062 ± 0.003	0.066 ± 0.007	0.119 ± 0.002
37	Geraniol	0.037 ± 0.009	0.135 ± 0.007	0.085 ± 0.006	0.033 ± 0.003	0.022 ± 0.001
38	4-Methyl anisole	0.073 ± 0.001	0.070 ± 0.005	0.085 ± 0.007	0.049 ± 0.010	0.042 ± 0.002
39	1-Octanol	0.247 ± 0.008	0.194 ± 0.007	0.100 ± 0.004	0.030 ± 0.002	0.031 ± 0.002
40	Propanoic acid	0.127 ± 0.001	0.042 ± 0.003	0.209 ± 0.002	0.055 ± 0.005	0.053 ± 0.001
41	Cyclohexanol	0.135 ± 0.001	0.125 ± 0.003	0.104 ± 0.008	0.161 ± 0.006	0.082 ± 0.002
42	L-Cysteine hydrochloride	0.095 ± 0.001	0.079 ± 0.005	0.091 ± 0.006	0.173 ± 0.008	0.205 ± 0.002
43	n-Decanoic acid	0.139 ± 0.001	0.071 ± 0.006	0.114 ± 0.005	0.083 ± 0.011	0.082 ± 0.002
44	Citronellal	0.317 ± 0.001	0.207 ± 0.007	0.156 ± 0.007	0.065 ± 0.005	0.037 ± 0.001
45	Azadirachtin	0.072 ± 0.044	0.070 ± 0.010	0.163 ± 0.019	0.193 ± 0.013	0.123 ± 0.027
46	3-Hexanol	0.089 ± 0.018	0.104 ± 0.032	0.156 ± 0.014	0.222 ± 0.031	0.285 ± 0.030
47	Dodecane	0.099 ± 0.013	0.139 ± 0.027	0.181 ± 0.009	0.234 ± 0.031	0.195 ± 0.025
48	Tetradecane	0.189 ± 0.018	0.240 ± 0.013	0.163 ± 0.029	0.312 ± 0.029	0.224 ± 0.025
49	Hexadecane	0.167 ± 0.055	0.066 ± 0.014	0.108 ± 0.027	0.220 ± 0.023	0.344 ± 0.053
50	Octadecane	0.140 ± 0.066	0.091 ± 0.050	0.152 ± 0.036	0.180 ± 0.041	0.253 ± 0.033
51	Eicosane	0.116 ± 0.036	0.174 ± 0.035	0.268 ± 0.064	0.264 ± 0.012	0.324 ± 0.031
52	Docosane	0.132 ± 0.045	0.098 ± 0.021	0.253 ± 0.043	0.291 ± 0.037	0.255 ± 0.035
53	Benzaldehyde	0.105 ± 0.050	0.187 ± 0.035	0.173 ± 0.015	0.202 ± 0.031	0.218 ± 0.021
54	Eugenol	0.066 ± 0.006	0.162 ± 0.022	0.162 ± 0.051	0.139 ± 0.036	0.224 ± 0.024

**Table 4:** Multichannel olfactometric bioassay analysis of Ixodidae tick *Haemaphysalis darjeeling* against the compounds with binomial test analysis between positive and negative responses.

Sl No.	Name of Compound	Positive Responses	Negative Responses	No Response	Total Specimen Count	Binomial *p* Value at 0.05 Level of Significance	Significant/Non-Significant
1	4-Nitrophenol	5.7 ± 0.9	1.7 ± 0.9	22.7 ± 1.7	30	0.2266	Non-significant
2	Methyl salicylate	20.7 ± 0.9	2 ± 0.6	7.3 ± 1.2	30	0.0001	Significant
3	Nonanoic acid	16.3 ± 0.9	3.3 ± 0.9	10.3 ± 1.3	30	0.0022	Significant
4	Adenine	16.7 ± 1.5	4 ± 0.6	9.3 ± 1.3	30	0.0059	Significant
5	Guanine	14.7 ± 1.2	3.3 ± 0.7	12 ± 1.7	30	0.0154	Significant
6	Hematin porcine	20 ± 1.7	2 ± 0.6	8 ± 2.1	30	0.0001	Significant
7	Uric acid	19.3 ± 1.2	2.7 ± 0.3	8 ± 1.5	30	0.0004	Significant
8	Xanthine	5.3 ± 0.9	2.3 ± 1.5	22.3 ± 2.3	30	0.2266	Non-significant
9	2,6-Dichlorophenol	15.3 ± 0.7	3.7 ± 1.2	11 ± 0.6	30	0.0096	Significant
10	1-Octen-3-ol	22.7 ± 1.5	2 ± 1	5.3 ± 1.7	30	0.0000	Significant
11	2-Ethylhexanoic acid	14 ± 0.6	2.7 ± 0.9	13.3 ± 0.7	30	0.0021	Significant
12	2-Nitrophenol	8.3 ± 0.7	2.3 ± 0.7	19.3 ± 0.7	30	0.0547	Non-significant
13	Acetone	7 ± 1.2	4 ± 0.6	19 ± 1.5	30	0.2744	Non-significant
14	Tiglic aldehyde	5.3 ± 0.9	15 ± 1.2	9.7 ± 0.9	30	0.0207	Significant
15	Ammonia	24.7 ± 1.2	2.7 ± 0.9	2.7 ± 0.3	30	0.0000	Significant
16	p-Tolualdehyde	18.7 ± 0.9	1.7 ± 1.2	9.7 ± 1.5	30	0.0002	Significant
17	Benzoic acid	13.3 ± 0.3	3 ± 1	13.7 ± 1.3	30	0.0106	Significant
18	Butyric acid	4 ± 0.6	18.7 ± 1.8	7.3 ± 1.2	30	0.0022	Significant
19	Carbon dioxide	19 ± 1.2	1.7 ± 1.7	9.3 ± 2	30	0.0000	Significant
20	Heptanoic acid	16.3 ± 1.5	4 ± 1.5	9.7 ± 3	30	0.0059	Significant
21	Hexanal	5.7 ± 0.9	1.7 ± 0.9	22.7 ± 1.3	30	0.2266	Non-significant
22	Hexanoic acid	17.3 ± 0.9	4.3 ± 1.3	8.3 ± 1.5	30	0.0036	Significant
23	Isobutyric acid	6 ± 1.5	12 ± 1.5	12 ± 3.1	30	0.1189	Non-significant
24	Mucochloric acid	8.7 ± 0.9	2.3 ± 1.2	19 ± 1	30	0.1133	Non-significant
25	Pentanoic acid	17.3 ± 0.3	3 ± 0.6	9.7 ± 0.7	30	0.0013	Significant
26	Pyruvate	23.3 ± 0.9	3.7 ± 0.9	3 ± 1.5	30	0.0002	Significant
27	Hexane	5.3 ± 1.2	1 ± 0.6	23.7 ± 0.7	30	0.1094	Non-significant
28	cis-3-Hexanoic acid	13 ± 1.2	3.3 ± 1.3	13.7 ± 2.4	30	0.0106	Significant
29	(R)-(+) Limonene	8 ± 0.6	2 ± 1	20 ± 1.2	30	0.0547	Non-significant
30	n-Octanoic acid	12.7 ± 1.5	4.7 ± 0.3	12.7 ± 1.5	30	0.0717	Non-significant
31	Ethyl Butyrate	10.3 ± 1.8	4.3 ± 1.3	15.3 ± 0.7	30	0.0898	Non-significant
32	Citral	5.3 ± 0.9	7.3 ± 0.9	17.3 ± 0.9	30	0.3872	Non-significant
33	Butyl butyrate	4.3 ± 0.7	3.7 ± 1.2	22 ± 0.6	30	0.6367	Non-significant
34	Isopropyl acetate	12.3 ± 0.9	2 ± 1	15.7 ± 1.5	30	0.0065	Significant
35	β-Caryophyllene	4.7 ± 0.9	2.7 ± 1.5	22.7 ± 0.7	30	0.5000	Non-significant
36	(R)-(+)α-pinene	7.7 ± 1.8	2.7 ± 0.3	19.7 ± 1.5	30	0.1719	Non-significant
37	Geraniol	5 ± 1.2	19.3 ± 1.5	5.7 ± 1.2	30	0.0033	Significant
38	4-Methyl anisole	12.3 ± 0.7	3.3 ± 0.9	14.3 ± 0.3	30	0.0176	Significant
39	1-Octanol	11.3 ± 1.2	1 ± 0.6	17.7 ± 0.9	30	0.0032	Significant
40	Propanoic acid	19.3 ± 1.2	2 ± 0.6	8.7 ± 1.5	30	0.0001	Significant
41	Cyclohexanol	14.3 ± 0.9	3 ± 0.6	12.7 ± 1.2	30	0.0064	Significant
42	L-cysteine hydrochloride	6.3 ± 1.2	2.7 ± 0.7	21 ± 0.6	30	0.2539	Non-significant
43	N-Decanoic acid	9.3 ± 0.9	2.3 ± 1.5	18.3 ± 0.7	30	0.0327	Significant
44	Citronellal	1.3 ± 0.9	25.3 ± 1.2	3.3 ± 0.3	30	0.0000	Significant
45	Azadirachtin	14 ± 1.5	14 ± 1.5	2 ± 3.1	30	0.0384	Significant
46	3-Hexanol	18.3 ± 0.9	1.7 ± 0.7	10 ± 1	30	0.0002	Significant
47	Dodecane	14.7 ± 0.7	2.7 ± 0.3	12.7 ± 0.9	30	0.0064	Significant
48	Tetradecane	18 ± 1.5	3 ± 1	9 ± 2.5	30	0.0007	Significant
49	Hexadecane	16.3 ± 1.5	2.7 ± 0.3	11 ± 1.2	30	0.0022	Significant
50	Octadecane	16 ± 1.5	4 ± 1.2	10 ± 1.7	30	0.0059	Significant
51	Eicosane	13 ± 0.6	3 ± 1.5	14 ± 1.5	30	0.0106	Significant
52	Docosane	10 ± 1	3.3 ± 0.9	16.7 ± 1.2	30	0.0461	Significant
53	Benzaldehyde	4 ± 0.6	17.3 ± 1.2	8.7 ± 1.2	30	0.0096	Significant
54	Eugenol	3.7 ± 0.9	23.3 ± 1.5	3 ± 2.1	30	0.0002	Significant

**Table 5:** Y-tube bioassay of the compounds in *H. darjeeling*.

**SL No.**	**Name of Organic Compound**	**Time Taken by the Tick to Reach the Arm (in Minutes)**	**Type of Response**	**Number of Specimens**
1	Ammonia	2.5	Positive	30
2	Pyruvate	2.9	Positive	30
3	1-Octen-3-ol	3.3	Positive	30
4	Hematin porcine	3.4	Positive	30
5	p-Tolualdehyde	3.5	Positive	30
6	Methyl salicylate	3.5	Positive	30
7	Uric acid	3.8	Positive	30
8	Tetradecane	3.9	Positive	30
9	Carbon dioxide	4.0	Positive	30
10	Propanoic acid	4.1	Positive	30
11	3-Hexanol	4.4	Positive	30
12	Hexanoic acid	4.5	Positive	30
13	Adenine	4.5	Positive	30
14	2,6-Dichlorophenol	4.7	Positive	30
15	Hexadecane	4.8	Positive	30
16	Heptanoic acid	4.9	Positive	30
17	Pentanoic acid	5.1	Positive	30
18	Octadecane	5.3	positive	30
19	Guanine	5.3	Positive	30
20	Nonanoic acid	5.5	Positive	30
**Sl. No.**	**Name of the Organic Compounds**	**Time Taken by the Tick to Reach the Vacant Arm (in Minutes)**	**Type of Responses**	**Number of Total Specimens**
1.	Citronellal	2.3	Negative	30
2.	Eugenol	2.9	Negative	30
3.	Butyric acid	3.3	Negative	30
4.	Geraniol	3.4	Negative	30
5.	Benzaldehyde	4.0	Negative	30
6.	Tiglic aldehyde	4.2	Negative	30

## Data Availability

Raw data are partly available in Zenodo (https://doi.org/10.5281/zenodo.14849790 (accessed on 5 May 2025)); the remaining data are available upon request.

## Supplementary Materials

The following supporting information can be downloaded at: https://www.mdpi.com/article/10.3390/bios15060358/s1, Figure S1: Dorsal view of the tick synganglion showing different nerve connections and the location where the recording electrode has been inserted in the scutum region. (PeN1-4: pedal nerves 1-4; PgS: periganglionic sheath; Pc: prothocerebral lobe). Figure S2: An overview of the eight-arm olfactometer. Figure S3: Representative raw data of ESG spectra measured in mV of few stimulated volatile organic compounds. Figure S4: A, B. Mean normalized electroscutumography (ESG) responses in *Haemaphysalis darjeeling* with surgically removed Haller’s organ to volatile organic compounds at 0.001 and 0.01% concentrations. Figure S5: C, D. Mean normalized electroscutumography (ESG) responses in *Haemaphysalis darjeeling* with surgically removed Haller’s organ to volatile organic compounds at 0.1 and 1% concentrations. Figure S6: E. Mean normalized electroscutumography (ESG) responses in *Haemaphysalis darjeeling* with surgically removed Haller’s organ to volatile organic compounds at 2% concentration. Table S1: Display of the corresponding mean differences and variances of the ESG data.
