# Supplementary material for: Sensing of Volatile Organic Compounds by Haller’s Structure in Ixodidae Tick: Electroscutumography and Olfactometric Bioassay
Source: Biosensors (Basel). 2025 Jun 4;15(6):358. doi: 10.3390/bios15060358 (PMC12191319; doi:10.3390/bios15060358)
Supplement: Supplementary file 1 [file biosensors-15-00358-s001.zip › biosensors-3566976-supplementary.pdf]

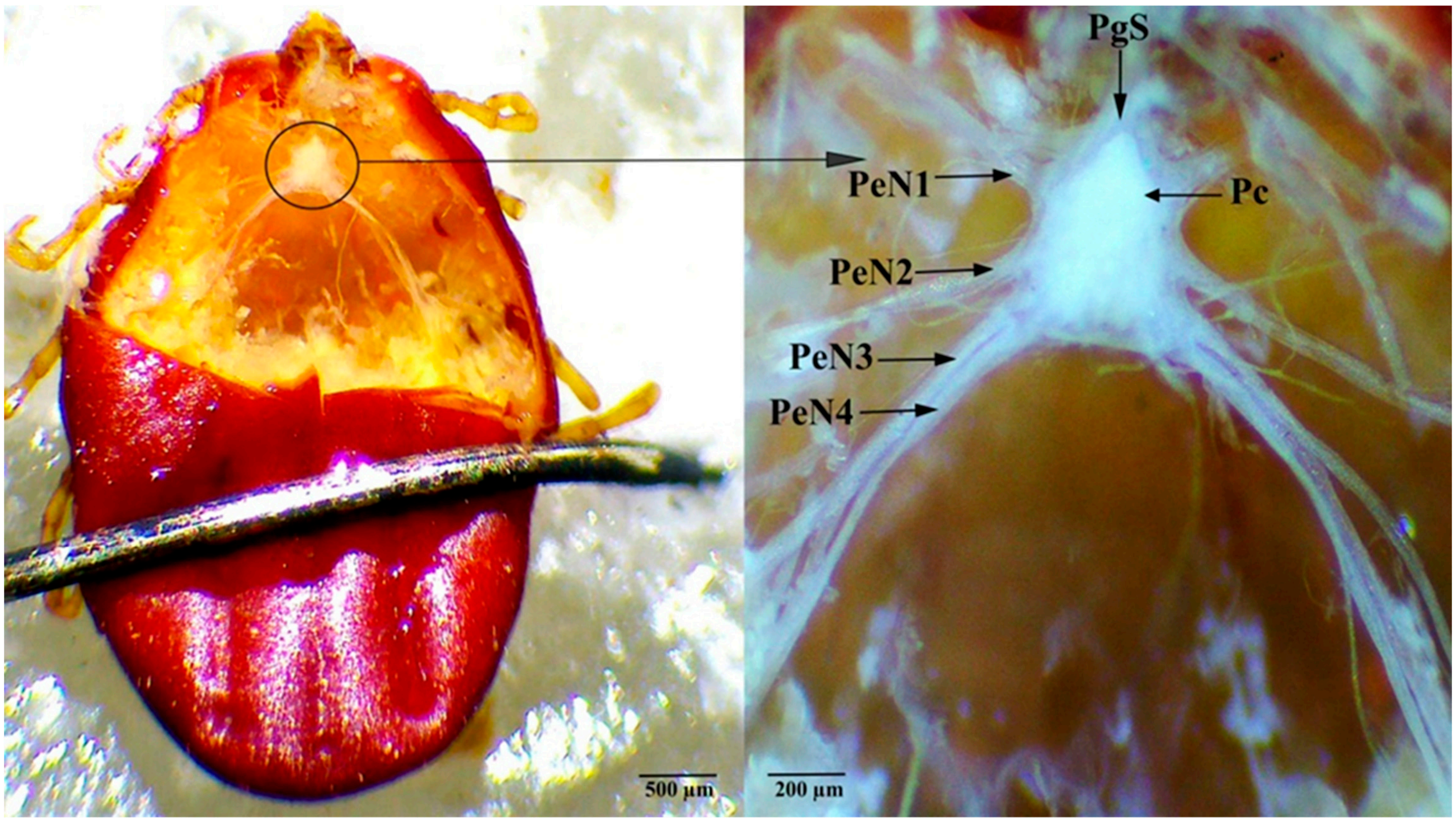

Figure S1. Dorsal view of the tick synganglion showing different nerve connections and the location where the recording electrode has been inserted in the scutum region. (PeN1-4: pedal nerves 1-4; PgS: periganglionic sheath; Pc: prothocerebral lobe).

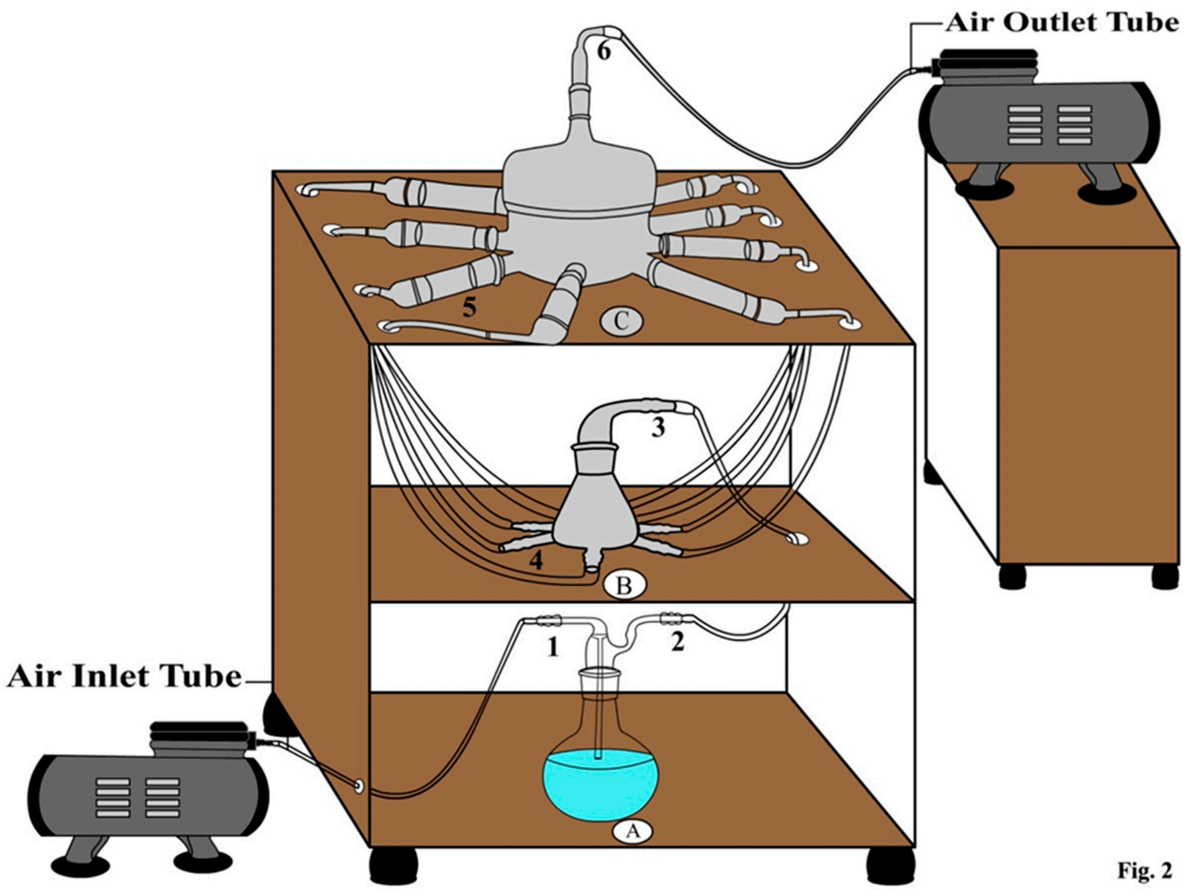

Figure S2. An overview of the eight-arm olfactometer.

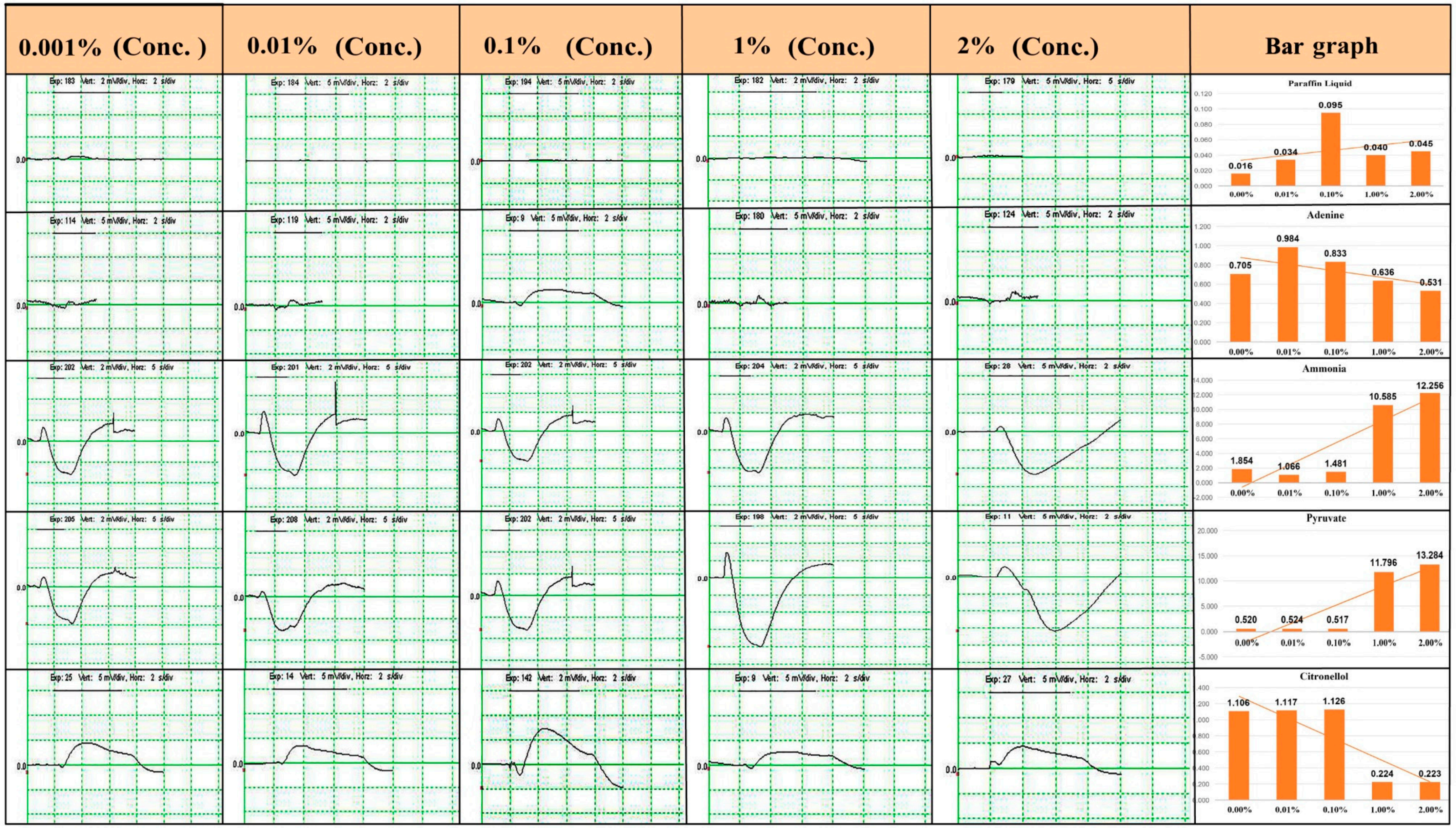

Fig.S1

Figure S3 Representative raw data of ESG spectra measured in mV of few stimulated volatile organic compounds.

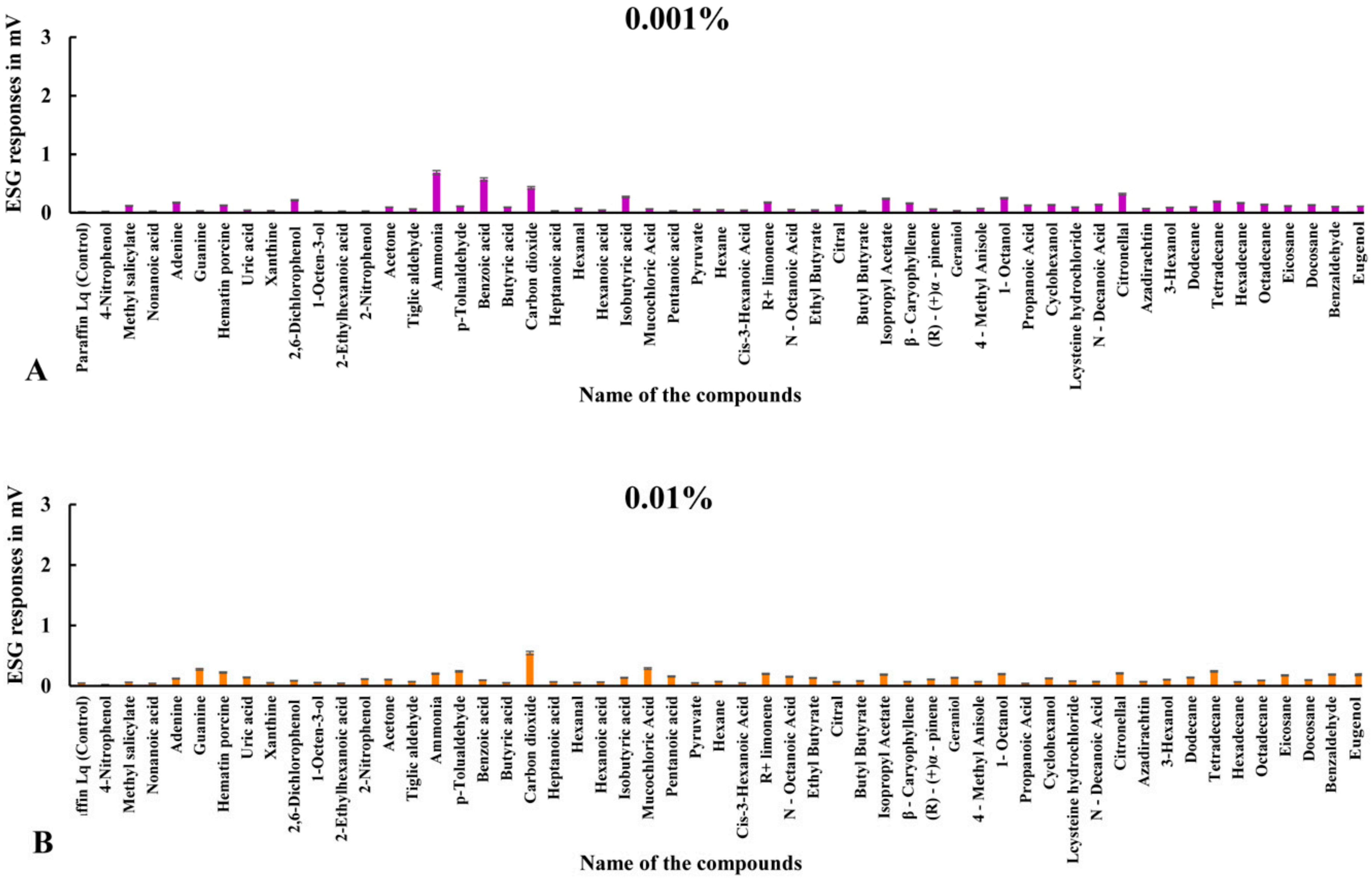

Figure S4 A,B. Mean normalized electroscutumography (ESG) responses in *Haemaphysalis darjeeling* with surgically removed Haller's organ to volatile organic compounds at 0.001 and 0.01 % concentrations.

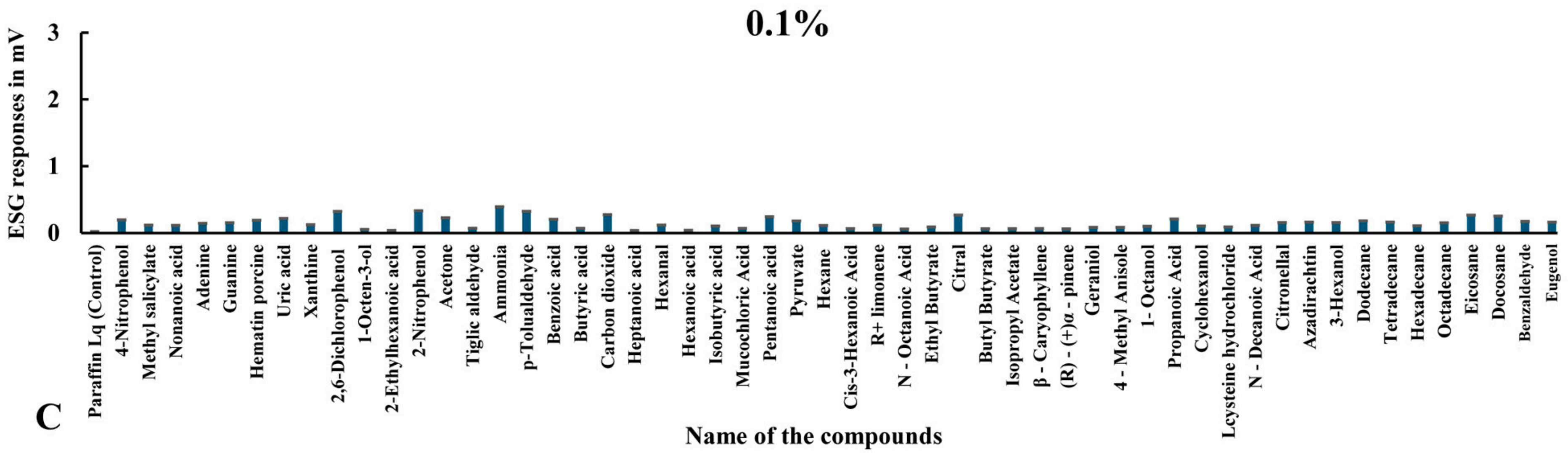

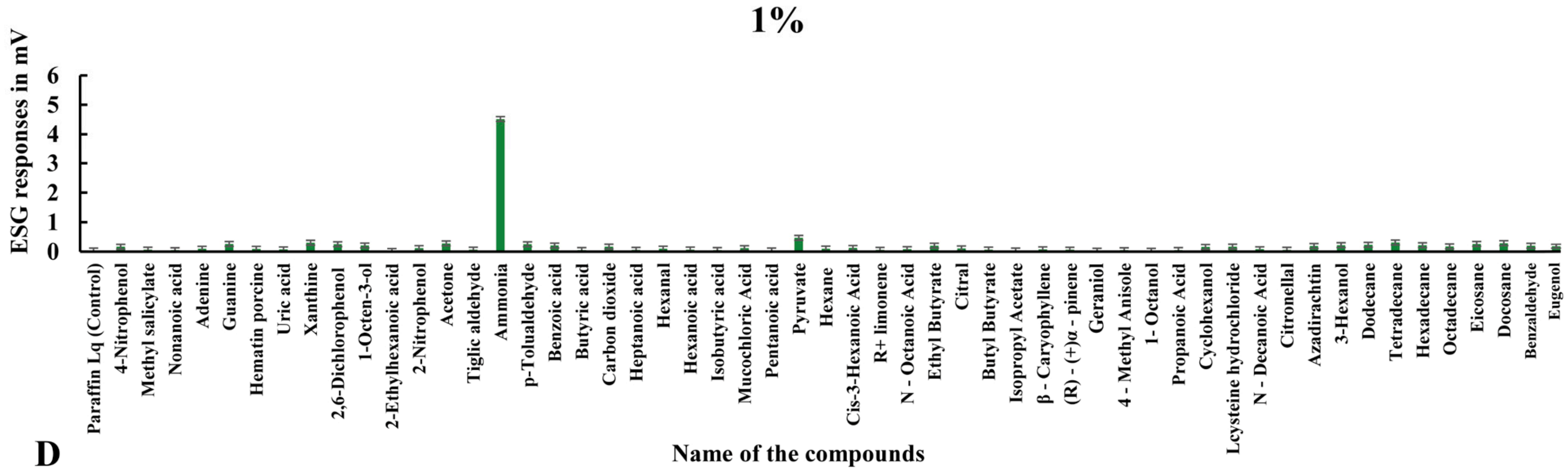

Figure S5 C,D. Mean normalized electroscutumography (ESG) responses in *Haemaphysalis darjeeling* with surgically removed Haller's organ to volatile organic compounds at 0.1 and 1 % concentrations.

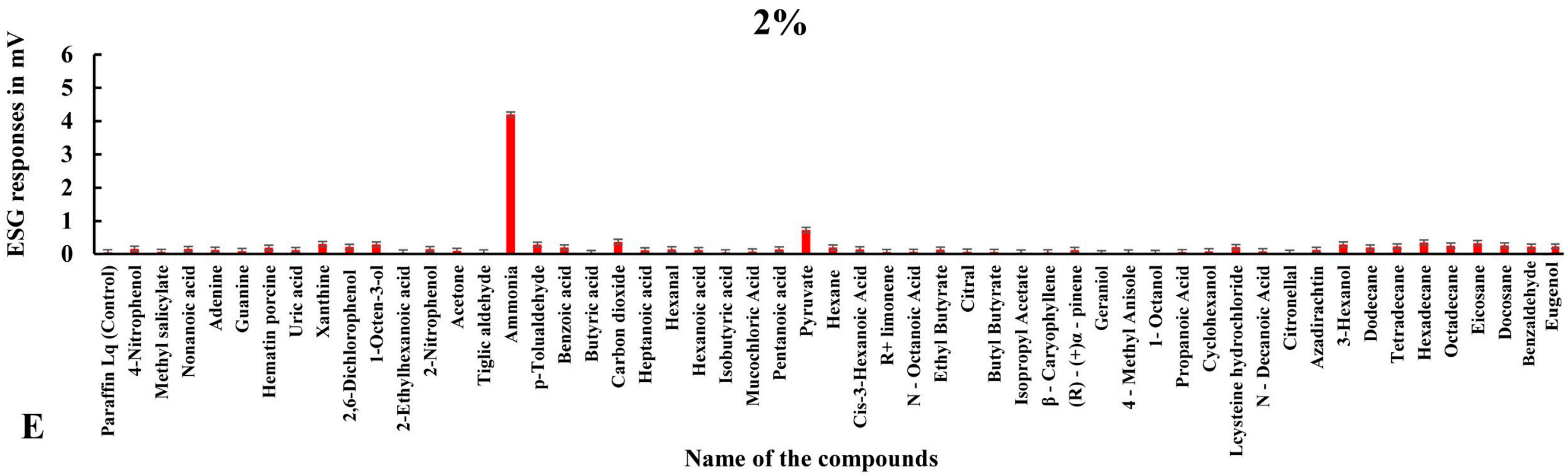

Figure S6 E. Mean normalized electroscutumography (ESG) responses in *Haemaphysalis darjeeling* with surgically removed Haller's organ to volatile organic compounds at 2 % concentration.

Table S1 Display of the corresponding mean differences and variances of the ESG data.
A: Group mean under five concentrations in Intact condition

ESG values under different concentrations (*w/v*) in intact condition

0.001%	0.01%	0.1%	1%	2%
0.082	0.071	0.074	0.619	0.707
0.512	0.433	0.480	0.416	0.598
0.129	0.131	0.137	0.334	0.463
0.705	0.984	0.833	0.636	0.531
0.697	0.483	0.637	0.725	0.852
0.509	0.779	0.655	0.761	0.794
0.311	0.616	0.463	0.688	0.730
0.290	0.408	0.337	0.561	0.530
1.340	0.600	0.956	1.189	0.977
0.180	0.157	0.179	0.958	1.020
0.139	0.244	0.189	0.235	0.191
0.228	0.444	0.367	0.536	0.505
0.240	0.412	0.340	0.477	0.995
0.588	0.495	0.553	0.174	0.479
1.854	1.066	1.481	10.58	12.25
0.666	1.261	0.958	1.072	1.264
1.998	0.630	1.331	0.986	0.980
0.415	0.245	0.310	0.240	0.467
0.675	0.726	1.252	0.966	1.589
0.228	0.182	0.205	0.501	0.406
0.401	0.112	0.273	0.639	0.697
0.137	0.655	0.371	0.350	0.351
1.385	0.785	1.074	0.214	0.175
0.526	0.381	0.403	0.598	0.537
0.626	0.538	0.714	0.856	0.544
0.520	0.524	0.517	11.79	13.28
0.333	0.474	0.394	0.673	0.466
0.110	0.223	0.184	0.620	0.466
0.951	0.399	0.646	0.428	0.345
0.360	0.680	0.507	0.677	0.496
0.275	0.661	0.472	0.874	0.849
0.428	0.392	0.413	0.322	0.281
0.111	0.260	0.185	0.462	0.554
0.918	1.047	0.987	0.444	0.438
0.659	0.382	0.539	0.203	0.227
0.472	0.402	0.452	0.367	0.365
0.183	0.572	0.373	0.890	0.776
0.256	0.225	0.278	0.172	0.118
1.012	0.880	0.967	0.342	0.492
0.667	0.974	0.847	0.277	0.456
0.693	0.714	0.702	0.721	1.031

0.431	0.382	0.422	0.741	0.988
0.715	0.805	0.781	0.554	0.927
1.106	1.117	1.126	1.04	0.823
0.322	0.680	0.510	0.405	0.589
0.259	0.288	0.426	0.606	0.899
0.467	0.589	0.674	0.737	0.856
0.494	0.672	0.584	0.916	1.23
0.426	0.491	0.583	0.738	0.952
0.485	0.372	0.603	0.748	0.637
0.283	0.485	0.596	0.894	0.799
0.596	0.754	0.695	0.834	1.17
0.315	0.485	0.369	0.684	0.593
0.583	0.495	0.795	0.848	0.957
**Group Mean**	**Group Mean**	**Group Mean**	**Group Mean**	**Group Mean**
0.542	0.541	0.577	1.006	1.124

B: One- way ANOVA between five concentrations in intact condition

**Source**	**Sum of Square *(SS)***	***df***	**Mean Square**	**F Statistic**	**P-value**
Between groups	17.3475	4	4.3369	*F* = 1.49902	P= 0.07022
Error (within groups)	524.2958	265	1.9785		
Total	541.6432	269	2.0135		

The f-ratio value is 1.49902. The p-value is 0.07022

The result is not significant at $p < 0.05$.

C: Post Hoc Turkey HSD test

**Pair**	**Difference**	**SE**	**Q**	**Lower CI**	**Upper CI**	**Critical Mean**	**p-value**
x1-x2	0.002574	0.05136	0.05012	-0.1969	0.2021	0.1995	1
x1-x3	0.02211	0.05136	0.4306	-0.1774	0.2216	0.1995	0.9981
x1-x4	0.09661	0.05136	1.8812	-0.1029	0.2961	0.1995	0.6726
x1-x5	0.1014	0.05136	1.9753	-0.09804	0.3009	0.1995	0.6303
x2-x3	0.02468	0.05136	0.4807	-0.1748	0.2242	0.1995	0.9971
x2-x4	0.09919	0.05136	1.9314	-0.1003	0.2987	0.1995	0.6502
x2-x5	0.104	0.05136	2.0255	-0.09546	0.3035	0.1995	0.6074
x3-x4	0.0745	0.05136	1.4507	-0.125	0.274	0.1995	0.8433
x3-x5	0.07933	0.05136	1.5448	-0.1201	0.2788	0.1995	0.8104

x4-x5	0.004833	0.05136	0.09411	-0.1946	0.2043	0.1995	1

Group	x2	x3	x4	x5
x1	0.0026	0.022	0.097	0.1
x2	0	0.025	0.099	0.1
x3	0.025	0	0.075	0.079
x4	0.099	0.075	0	0.0048

D: Test results of One-way ANOVA across five different concentrationsamong intact condition and after removing the Haller's organ condition

**Concentration**	**Source**	***df***	**Sum of Square**	**Mean Square**	**F Statistic**	**P-value**
0.001%	Groups (between groups)	1	4.6588	4.6588	52.516	7.21E-11
	Error (within groups)	106	9.4035	0.08871		
	Total	107	14.0623	0.1314		

Since p-value<α, H_o is rejected.
Some of the groups' averages consider being not equal.

**Concentration**	**Source**	***df***	**Sum of Square**	**Mean Square**	**F Statistic**	**P-value**
0.01%	Groups (between groups)	1	4.7046	4.7046	116.0991	1.11E-16
	Error (within groups)	106	4.2954	0.04052		
	Total	107	9	0.08411		

Since p-value<α, H_o is rejected.
Some of the groups' averages consider being not equal.

**Concentration**	**Source**	***df***	**Sum of Square**	**Mean Square**	**F Statistic**	**P-value**
0.1%	Groups (between groups)	1	4.9601	4.9601	93.515	-2.22E-16
	Error (within groups)	106	5.6223	0.05304		
	Total	107	10.5824	0.0989		

Since p-value<α, H_o is rejected.
Some of the groups' averages consider being not equal.

**Concentration**	**Source**	***df***	**Sum of Square**	**Mean Square**	**F Statistic**	**P-value**
1%	Groups (between groups)	1	16.5299	16.5299	7.3374	0.007876
	Error (within groups)	106	238.8012	2.2528		
	Total	107	255.3311	2.3863		

Since p-value<α, H0 is rejected.
Some of the groups' averages consider being not equal.

**Concentration**	**Source**	***df***	**Sum of Square**	**Mean Square**	**F Statistic**	**P-value**
2%	Groups (between groups)	1	21.6581	21.6581	7.554	0.00704

	Error (within groups)	106	303.9138	2.8671		
	Total	107	325.572	3.0427		
Since p-value<α, H_o is rejected.<br>Some of the groups' averages consider being not equal.						
